# Prevalence and factors associated with depression, anxiety and post-traumatic stress disorder among healthcare workers from sub-Saharan Africa: systematic review

**DOI:** 10.1192/bjo.2025.10818

**Published:** 2025-09-08

**Authors:** Ezra Kipngetich Too, Peninah Wachira, Solomon Njenga, Sabina Adhiambo Odero, Eunice Ndirangu-Mugo, Amina Abubakar

**Affiliations:** Institute for Human Development, Aga Khan University, Nairobi, Kenya; Faculty of Medicine and Health Sciences, University of Antwerp, Antwerp, Belgium; School of Nursing and Midwifery, Aga Khan University, Nairobi, Kenya

**Keywords:** Depression, anxiety, PTSD, healthcare workers, sub-Saharan Africa

## Abstract

**Background:**

Depression, anxiety and post-traumatic stress disorder (PTSD) are prevalent among healthcare workers (HCWs), including those from sub-Saharan Africa (SSA). However, there are limited summary data on the burden and factors associated with these disorders in this region. We conducted this systematic review (registration no. CRD42022349136) to fill this gap.

**Aims:**

The aim of this review was to systematically summarise the available evidence on the prevalence and factors associated with depression, anxiety and PTSD, or their symptoms, among HCWs from SSA.

**Method:**

We searched African Index Medicus, African Journals Online, CINAHL, PsycINFO and PubMed for articles published, from database inception to 15 February 2024. The keywords used in the search were ‘depression/anxiety/PTSD’, ‘healthcare workers’, ‘SSA’ and their variations.

**Results:**

Sixty-nine studies met our inclusion criteria, most of which (*n* = 55, 79.7%) focused on the burden of these disorders during the COVID-19 pandemic. Across studies, wide-ranging prevalence estimates of depressive (2.1–75.7%), anxiety (4.8–96.5%) and PTSD symptoms (11.7–78.3%) were reported. These disorders appear to have been heightened during the COVID-19 pandemic. Several sociodemographic, health-related, COVID-19-related and work-related factors were reported to either increase or lower the risk of these disorders among HCWs from SSA.

**Conclusions:**

The burden of depression, anxiety and PTSD among HCWs from SSA is high and appears to have been worsened by the COVID-19 pandemic. The correlates of these disorders among HCWs from this region are multifactorial. A multi-component intervention could contribute to addressing the burden of mental disorders among HCWs from this region.

Healthcare workers (HCWs) are among high-risk subpopulations with a disproportionate burden of mental disorders compared with the general population.^1,2^ Depression, anxiety and post-traumatic stress disorder (PTSD) are particularly prevalent in this population.^3^ For instance, evidence from 65 studies included in a systematic review to assess the prevalence of these disorders among a total of 97 333 HCWs globally showed that the pooled prevalence of moderate depression, anxiety and PTSD was 21.7, 22.1 and 21.5%, respectively.^3^

Numerous factors contribute to the elevated burden of mental disorders among HCWs. The nature of their work, which often includes long working hours and working night shifts, with little time for social and personal life, contributes significantly to this burden.^4^ HCWs also frequently experience sleep problems such as sleep deprivation, insomnia and poor-quality sleep, which are associated with mental health problems.^5,6^ HCWs are also constantly exposed to human suffering and death in their line of work.^7,8^ They may also be frequently exposed to abuse, hostility and violence in their workplace.^9^ A higher risk of infection with infectious diseases also exacerbates the risk of mental disorders in this population.^10^ Occasionally being on the front lines dealing with mass casualty events, such as disasters and pandemics like COVID-19, also plays a significant role in the heightened risk of mental disorders in this population.^11,12^

In sub-Saharan Africa (SSA) the burden of mental disorders among HCWs is significant, and is even thought to be higher when compared with other regions. For instance, a systematic review that compared the pooled prevalence of depression among HCWs across regions reported the highest prevalence in Africa (82.4%), compared with pooled prevalence estimates of 33.4, 31.3 and 19.1% in North America, Europe and Asia, respectively.^13^ The burden of mental disorders is thought to be particularly high among sub-Saharan HCWs because they face additional challenges and risk factors such as huge staff shortages,^4^ high patient:health worker ratios,^4^ high workloads,^4^ under-resourced healthcare systems,^14^ low remuneration and disproportionately high disease burden, including highly infectious epidemics such as Ebola virus disease and HIV.^15^

Despite the high burden of mental disorders among HCWs in SSA, to our knowledge only two systematic reviews assessing these disorders in this region have been published.^16,17^ However, these reviews focused only on studies that were conducted during the COVID-19 pandemic, and consequently they lack data from the periods preceding and following the pandemic. The current review, therefore, has the additional value of providing a picture of mental disorders in this population preceding and following the COVID-19 pandemic. Additionally, while recognising the role of pandemics and other public health emergencies in heightening of mental health problems among HCWs,^11,12,18^ we also aim to highlight the potential impact of the COVID-19 pandemic on the mental health of HCWs in this region. While this evidence has been provided by the aforementioned reviews, these were conducted in the early stages of the pandemic and thus provide evidence from the early phase only. This review also includes studies from the later phases of the pandemic and utilises the data from these studies to strengthen the evidence on the impact of the pandemic on the mental health of HCWs.

Other existing relevant reviews have been global in nature,^3,10,13,19–21^ with the included studies originating mostly outside SSA, thus limiting their generalisability to this context. This review, therefore, aims to systematically summarise the available evidence on the prevalence and factors associated with mental disorders – specifically depression, anxiety and PTSD, or their symptoms – among HCWs from SSA.

## Method

### Review protocol and registration

Prior to the commencement of this systematic review, we developed the review protocol and registered it with the International Prospective Register of Systematic Reviews (PROSPERO) under registration no. CRD42022349136.

### Data sources

We conducted a literature search in the following electronic databases: African Index Medicus, African Journals Online, CINAHL, PsycINFO and PubMed. The search was for articles conducted from database inception to the final day of the database search. Initially, a search had been conducted and concluded on 17 June 2022. This search was later updated to 15 March 2023. A final update was conducted before submission, with the final database search occurring on 15 February 2024. The search was restricted to those studies published in English. All the identified articles were retrieved and uploaded to Rayyan software^22^ (release year: 2014, accessed via Windows; Rayyan Systems, Inc., Cambridge, Massachusetts, USA; https://www.rayyan.ai/) for data management. In addition, hand-searching was conducted through the reference lists of the included articles and related systematic reviews for additional relevant articles meeting the inclusion criteria of this review.

### Search strategy

We used a pre-developed search strategy to search the electronic databases mentioned above. The search strategy contained keywords including ‘depression/anxiety/PSTD’, ‘healthcare workers’ and ‘SSA’, combined using the Boolean Operator ‘AND’. Respective synonyms for these key words were combined using the Boolean operator ‘OR’. Supplementary File 1 provides the search strategy used in the PubMed database.

### Eligibility criteria

Table 1 provides the study eligibility criteria for this review.

**Table 1:** Study eligibility criteria

Inclusion criteria	Exclusion criteria
Studies on depression, anxiety and post-traumatic stress disorder	Studies on mental health disorders apart from depression, anxiety and post-traumatic stress disorder
Studies conducted among healthcare workers	Studies among populations other than healthcare workers, including medical students
Studies conducted in sub-Saharan Africa	Studies conducted outside sub-Saharan Africa
Cross-sectional studies, case-control studies and cohort studies	Reviews, qualitative studies, case studies, commentaries, conference papers, posters, study protocols, presentations, symposium abstracts, editorials, theses, letters to the editor, dissertations, books, book chapters, brief reports

### Screening of articles

P.W. and S.N. independently screened the articles for eligibility. E.K.T. later screened those articles that were identified in the updated search. Articles were screened on three levels, starting with screening for eligibility by title, then by abstract and finally by full text, during which ineligible studies were systematically excluded. Discrepancies were resolved through discussion and consensus with the other study co-authors.

### Data extraction

Data were extracted from the selected studies using a standardised, preprepared data extraction form. Data extraction began on 22 August 2022. Data extraction was dually and independently carried out by P.W. and S.N.; E.K.T. extracted data from the final updated articles. The extracted data include the following: (a) study details (name of first author, year of publication, country of origin and study design); (b) study participant characteristics (cadre of healthcare personnel recruited, sample size, sampling methods, age and gender); and (c) study outcomes (prevalence of depression, anxiety or PTSD, measurement tools used, cut-off scores applied (for screening tools), psychometric information of the tools used and factors associated with depression, anxiet, and PTSD among HCWs (alongside the reported measure of effect and precision estimate). Where the data of interest were not available in an article, the corresponding author was contacted to clarify or provide missing data. Papers where the contacted authors did not respond to the request were excluded from the review.

### Quality appraisal

The quality of the included articles was assessed using the Newcastle–Ottawa Scale (NOS) quality assessment scale adapted for cross-sectional studies. P.W. and S.N. equally shared the included studies and independently conducted quality appraisal. To reduce bias, E.K.T. conducted an independent appraisal of all the articles and compared scores with the individual scores from P.W. and S.N. Disagreements in quality ratings were resolved through discussion and consensus. The NOS uses a star scoring system with a maximum of nine points assigned to each article. This quality assessment tool assesses three main domains, including the ascertainment outcome of interest, the selection of study participants and the comparability of study groups. The scoring system awards five points for the selection domain, one point for the comparability domain and three points for the outcome domain. Scores of 0–4, 5–6, 7–8 and 9 are indicative of unsatisfactory, satisfactory, good and very good quality, respectively. In this review we used a score of ≥7 to indicate high-quality studies.

### Data synthesis

Because the significant heterogeneity of the measurement tools used across the included studies precluded a meta-analysis, we therefore conducted a narrative synthesis. We narratively summarised the prevalence estimates of depression, anxiety and PTSD among HCWs in SSA, as well as their associated factors, by the investigated outcome of interest. In this review, only those factors significantly associated with the outcomes of interest (depression, anxiety or PTSD) at *P* < 0.05 in the multivariable analysis were considered and extracted.

## Results

### Results of database search

**Fig. 1:**
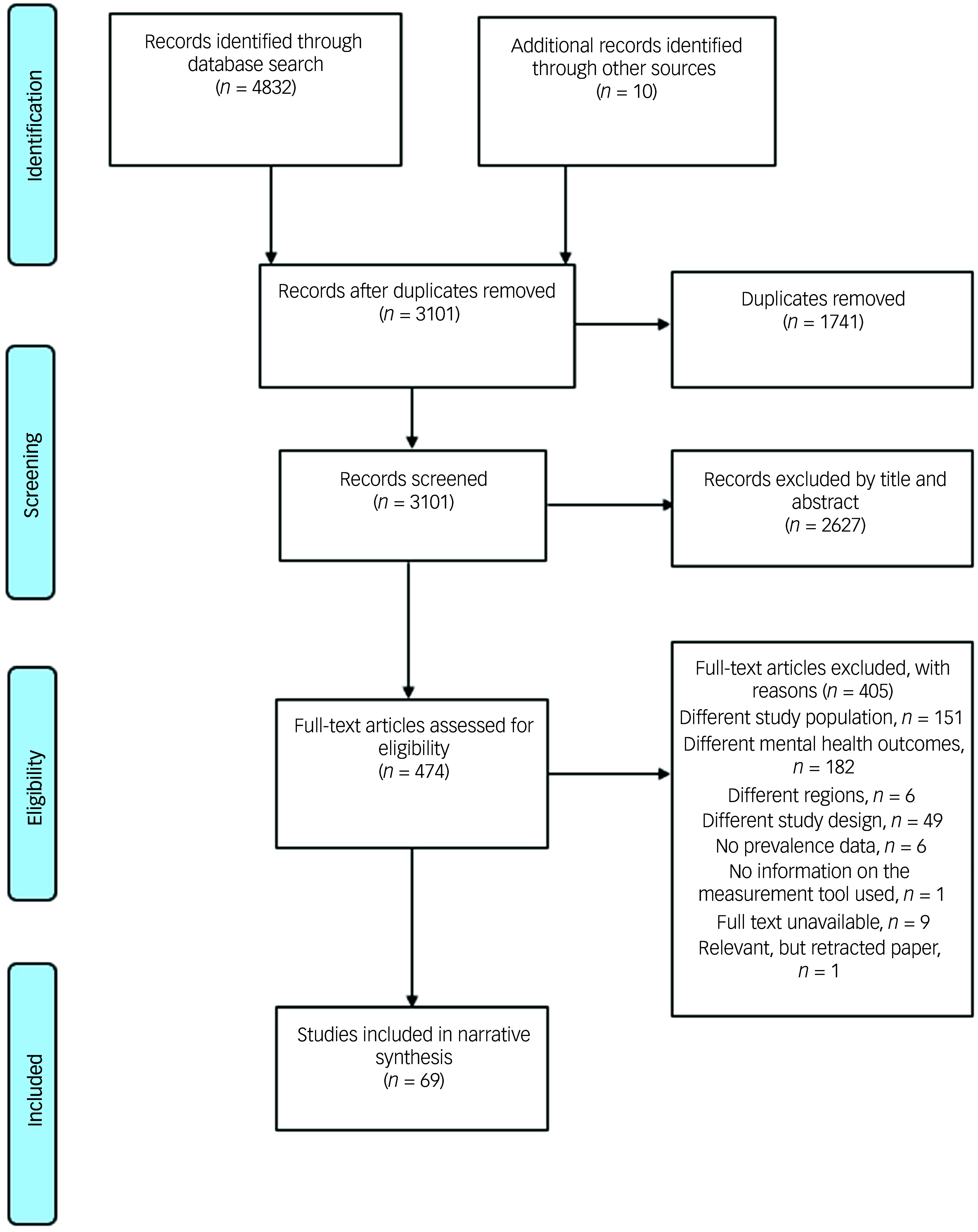
PRISMA flowchart for the systematic review process.

The database search yielded a total of 4832 articles (African Index Medicus, *n* = 44; African Journal Online, *n* = 369; CINAHL, *n* = 428; PsycINFO, *n* = 2141; and PubMed, *n* = 1850). Ten additional articles were retrieved from the reference lists of the included articles and related systematic reviews. Following the removal of duplicates and screening of articles based on the eligibility criteria, 69 articles were included in this review. Figure 1 shows the PRISMA flowchart for the systematic review process.

### Characteristics of included studies

**Fig. 2:**
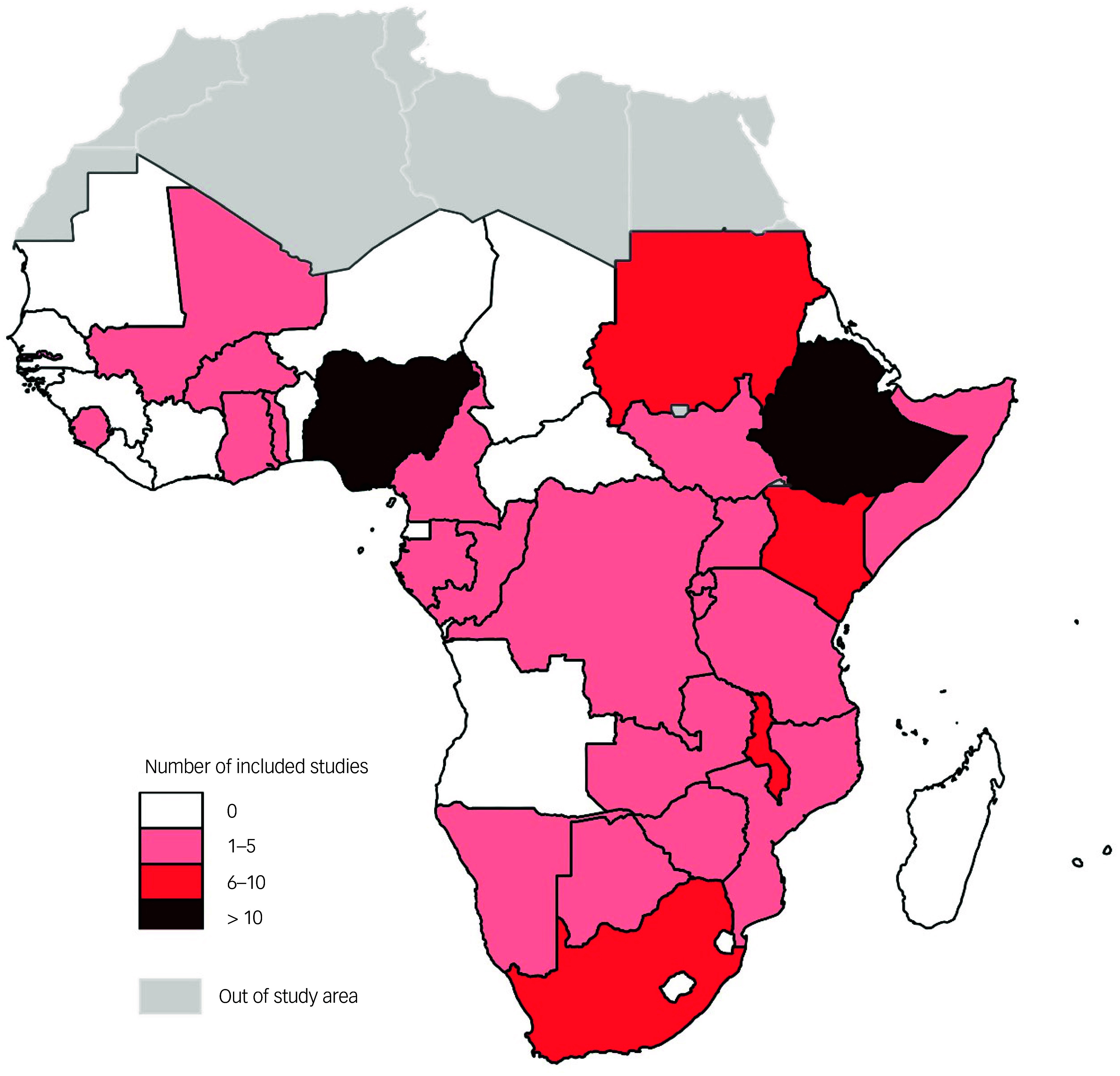
Distribution of the included studies.

Supplementary File 2 presents in detail the characteristics of the 69 studies included in this review; in summary, all included studies were cross-sectional in design. The included studies were conducted across 27 countries in SSA, with more than half (*n* = 37, 53.6%) being conducted in only three countries in SSA (Ethiopia, Nigeria and South Africa) (Fig. 2). Four of the included studies^23–26^ were multi-country studies that recruited HCWs from either 16,^24^ 13,^26^ 3^23^ or 2 countries.^25^ The total number of HCWs across these studies was 24 266. A comparison sample of 502 participants from the general population was also recruited in one of the included studies.^27^

Almost 80% of the included studies (*n* = 55, 79.7%) were studies assessing the burden of depression, anxiety or PTSD among HCWs in the context of the COVID-19 pandemic. The remaining 14 studies^4,7,8,14,24,28–36^ were conducted prior to the onset of the pandemic. Most of the included studies (*n* = 48, 69.6%) were conducted in health facilities while the remainder (*n* = 21, 30.4%) were conducted virtually through online platforms.

Over 70% of the included studies (*n* = 50, 72.5%) recruited multiple cadres of HCWs as study participants. In the remaining 19 studies, specific cadres of HCWs were recruited, including nurses recruited in 8 studies,^4,7,34,37–41^ doctors and nurses recruited in 3 studies,^42–44^ doctors in 5 studies,^30,32,45–47^ surgeons in 1 study,^24^ health extension workers in 1 study^31^ and midwives in 1 study.^8^ Study participants were recruited non-randomly in nearly half of the included studies (*n* = 28, 40.6%), whereas 42.0% (*n* = 29) of the included studies recruited their study participants using random sampling techniques. In the remaining 12 studies, information on the sampling methods was not reported.

Most of the included studies (*n* = 40, 58.0%) concurrently assessed at least two or more of the mental disorders of interest in this review (depression, anxiety and PTSD). In the remaining 29 studies, only 1 mental disorder was assessed, including depression in 14 studies,^4,14,24,26,28,29,31,33,36,44,48–51^ anxiety in 9 studies^7,8,25,30,38,52–55^ and PTSD in 7 studies.^35,39,41,56–59^ None of the included studies used a diagnostic interview to diagnose any of these disorders. Rather, all the included studies used mental health screening tools to identify the symptoms of each disorder. Information on the reliability and/or validity of these screening tools for use among HCWs from SSA is limited. Many of the included studies (*n* = 40, 57.1%) did not report any of this information. Where reported, only reliability (Cronbach’s *α*) was reported across studies (Table 2).

**Table 2:** Prevalence estimates of depression, anxiety and post-traumatic stress disorder (PTSD) among healthcare workers (HCWs) from sub-Saharan Africa (SSA)

			Depression				Anxiety				PTSD			
Author (year)	Period of data collection	Sample size	Measure used	Reliability	Cut-off score	Prevalence	Measure used	Reliability	Cut-off score	Prevalence	Measure used	Reliability	Cut-off score	Prevalence
Aliyu & Adeniyi, 2017^28^	Pre-COVID-19	734	DASS-21	NR	NR	11.4%	NA	NA	NA	NA	NA	NA	NA	NA
Belayneh et al, 2021^7^	Pre-COVID-19	415	NA	NA	NA	NA	HARS	NR	≥8	19.8%	NA	NA	NA	NA
Belete andAnbensaw, 2022^29^	Pre-COVID-19	252	PHQ-9	0.91	≥5	27.8%	NA	NA	NA	NA	NA	NA	NA	NA
Bernard et al, 2023^30^	Pre-COVID-19	629	NA	NA	NA	NA	GAD-7	0.72	NR	30.6%	NA	NA	NA	NA
Birhane et al, 2023^31^	Pre-COVID-19	584 rural HCWs; 581 urban HCWs	PHQ-9	NR	≥10	16.5% among rural HCWs; 8.9% among urban HCWs	NA	NA	NA	NA	NA	NA	NA	NA
Commander et al, 2020^24^	Pre-COVID-19	131	PHQ-9	NR	≥10	47.2%	NA	NA	NA	NA	NA	NA	NA	NA
Kim et al, 2019^14^	Pre-COVID-19	535	SRQ-20	0.79	≥8	6.7%	NA	NA	NA	NA	NA	NA	NA	NA
Mbanga et al, 2019^4^	Pre-COVID-19	143	PHQ-9	NR	≥4	62.2%	NA	NA	NA	NA	NA	NA	NA	NA
Muliira et al, 2015^8^	Pre-COVID-19	244	NA	NA	NA	NA	Death Distress Scale – Death Anxiety Subscale	NR	≥8, ≥19, ≥30	24.5, 54.5, and 20.1% mild, moderate and high anxiety, respectively	NA	NA	NA	NA
Naidoo et al, 2020^32^	Pre-COVID-19	150	PHQ-9	NR	≥10	21.3%	GAD-7	NR	≥10	20.0%	NA	NA	NA	NA
Obi et al, 2015^33^	Pre-COVID-19	309	Zung Self-Rating Depression Scale	NR	≥50	14.9%	NA	NA	NA	NA	NA	NA	NA	NA
Olabisi et al, 2021^34^	Pre-COVID-19	360	DASS-21	NR	≥10	38.5%	DASS-21	NR	≥8	46.6%	NA	NA	NA	NA
Olashore et al, 2018^35^	Pre-COVID-19	201	NA	NA	NA	NA	NA	NA	NA	NA	PCL-C	NR	≥6	18.4%
Pindar et al, 2015^36^	Pre-COVID-19	186	BDI-II	NR	≥10	10.8%	NA	NA	NA	NA	NA	NA	NA	NA
Agberotimi et al, 2020^27^	COVID-19 era	382 HCWs 502 from the general population	PHQ-9	0.87	NR	35.1% among HCWs 23.5% in thegeneral population; *P* < 0.01	GAD-7	0.81	NR	58.4% among HCWs 49.6% in the general population; *P* < 0.05	IES-R	0.82	NR	52.6% among HCWs 42.8% in the general population; *P* < 0.05
Ahmed et al, 2023^60^	COVID-19 era	167	DASS-21	NR	NR	22.8, 21.6, 18.0 and 7.2% mild, moderate, severe and very severe depression, respectively	DASS-21	NR	NR	25.8, 22.2, 13.2 and 12.6% mild, moderate, severe and very severe anxiety, respectively	NA	NA	NA	NA
Ali et al, 2021^37^	COVID-19 era	171	PHQ-9	0.87	≥10	45.9%	GAD-7	0.84	≥7	48.2%	IES-R	0.96	≥26	28.8%
Ali et al, 2022^45^	COVID-19 era	100	PHQ-9	0.91	≥10	64.3%	GAD-7	0.94	≥7	51.5%	IES-R	0.98	≥26	35.4%
Ariyo et al, 2021^61^	COVID-19 era	413	HAD	0.86	NR	26.4%	HAD	0.78	NR	25.2%	NA	NA	NA	NA
Arthur-Mensah et al, 2021^62^	COVID-19 era	38	BDI-II	NR	≥15	60.5%	STAI	NR	≥9.5 for state anxiety; ≥13.5 for trait anxiety	71.1% state anxiety 26.3% trait anxiety	NA	NA	NA	NA
Asnakew et al, 2021^56^	COVID-19 era	396	NA	NA	NA	NA	NA	NA	NA	NA	IES-R	0.92	≥24	55.1%
Assefa et al, 2021^23^	COVID-19 era	900	PHQ-4	NR	≥3	6.6% overall; 9.7% in Burkina Faso;6.0% in Ethiopia;4.0% in Nigeria	PHQ-4	NR	≥3	6.6% overall; 13.3% in Burkina Faso; 2.0% in Ethiopia; 4.3% in Nigeria	NA	NA	NA	NA
Ayalew et al, 2021^63^	COVID-19 era	387	DASS-21	0.95	≥5	50.1%	DASS-21	0.95	≥4	55.0%	NA	NA	NA	NA
Ayalew et al, 2022^57^	COVID-19 era	387	NA	NA	NA	NA	NA	NA	NA	NA	IES-R	0.94	≥24	56.8%
Bapolisi et al, 2022^64^	COVID-19 era	252	BDI-II	NR	≥20	40.0%	NA	NA	NA	NA	SARSQ	NR	≥90	16.0%
Bundi et al, 2023^65^	COVID-19 era	202	PHQ-9	NR	≥10	57.4%	GAD-7	NR	≥10	59.9%	NA	NA	NA	NA
Burnett-Zieman et al, 2023^66^	COVID-19 era	302	PHQ-9	0.83	≥10	9.0%	NA	NA	NA	NA	PC-PTSD-5	0.68	≥4	12.0%
Cenat et al, 2022^15^	COVID-19 era	254	NA	NA	NA	NA	HSCL	0.95	≥1.75	65.4%	PCL-5	0.97	≥33	30.8%
Chorwe-Sungani, 2022^38^	COVID-19 era	102	NA	NA	NA	NA	CAS	0.90	≥9	25.5%	NA	NA	NA	NA
Dawood et al, 2021^67^	COVID-19 era	312	DASS-21	NR	≥5	51.5%	DASS-21	NR	≥4	47.2%	IES-R	NR	≥24	13.7%
Duffton et al, 2023^46^	COVID-19 era	163	DASS-21	0.92	NR	22.1%	DASS-21	0.77	NR	43.6%	NA	NA	NA	NA
Elamin et al, 2020^68^	COVID-19 era	396	PHQ-9	0.93	≥5, 10, 15, 20	24.3, 20.2, 11.9 and 15.7% mild, moderate, moderately severe and severe depression, respectively	GAD-7	0.95	≥5, 10, 15	23.5, 46.5 and 16.7% mild, moderate and severe anxiety, respectively	IES-R	0.94	≥9, 26, 44	31.3, 24.7 and 14.6% mild, moderate and severe PTSD, respectively
Falade et al, 2022^69^	COVID-19 era	432	HAD	NR	≥8	66.5%	HAD	NR	≥8	96.5%	NA	NA	NA	NA
GebreEyesus et al, 2021^70^	COVID-19 era	322	PHQ-9	NR	≥10	25.8%	GAD-7	NR	≥8	36.0%	NA	NA	NA	NA
Hain et al, 2021^47^	COVID-19 era	96	PHQ-9	NR	≥10	35.6%	GAD-7	NR	≥10	23.3%	NA	NA	NA	NA
Hajure et al, 2021^71^	COVID-19 era	127	DASS-21	NR	≥10	43.3%	DASS-21	NR	≥8	51.2%	IES-R	NR	≥33	40.2%
Hassan et al, 2024^72^	COVID-19 era	385	PHQ-9	NR	NR	35.3 and 31.4% mild and moderate depression, respectively	GAD-7	NR	NR	29.6% mild anxiety	NA	NA	NA	NA
Human et al, 2023^58^	COVID-19 era	120	NA	NA	NA	NA	NA	NA	NA	NA	PCL-5	NR	≥31	11.7%
Ibigbami et al, 2022^42^	COVID-19 era	434	PHQ-9	0.83	≥10	6.7% moderate to severe depression	GAD-7	0.86	≥10	3.2% moderate to severe anxiety	NA	NA	NA	NA
Idrees & Bashir, 2023^73^	COVID-19 era	133	HAD	NR	NR	44.0%	HAD	NR	NR	47.0%	NA	NA	NA	NA
Jemal et al, 2022^74^	COVID-19 era	816	DASS-21	0.78	≥9	75.7%	DASS-21	0.86	≥7	79.0%	NA	NA	NA	NA
Kabunga & Okalo, 2021^39^	COVID-19 era	601	NA	NA	NA	NA	NA	NA	NA	NA	PCL-C	0.86	≥45	61.7%
Kibret et al, 2020^52^	COVID-19 era	305	NA	NA	NA	NA	GAD-7	0.92	≥5	62.9%	NA	NA	NA	NA
Kwobah et al, 2021^75^	COVID-19 era	957	PHQ-9	NR	NR	32.1%	GAD-7	NR	NR	36.1%	PC-PTSD (DSM-V)	NR	≥3	64.7%
Mc Magh et al, 2023^76^	COVID-19 era	330	PHQ-2	NR	≥3	13.6%	GAD-7 CAS	NR NR	≥5 ≥9	47.3% 4.8%	NA	NA	NA	NA
Mekonen et al, 2021^40^	COVID-19 era	293	DASS-21	NR	≥10	55.3%	DASS-21	NR	≥8	69.6%	NA	NA	NA	NA
Mokogwu, 2021^77^	COVID-19 era	32	PHQ-9	NR	≥5	12.5% mild depression	GAD-7	NR	≥5	9.4% mild anxiety	NA	NA	NA	
Mulatu et al, 2021^78^	COVID-19 era	420	PHQ-9	0.86	≥10	20.2%	GAD-7	0.88	≥9	21.9%	IES-R	0.83	≥33	15.5%
Nguepy Keubo et al, 2020^79^	COVID-19 era	292	HAD	0.74	≥11	43.5%	HAD	0.70	≥11	42.2%	NA	NA	NA	NA
Ofori et al, 2021^80^	COVID-19 era	272	DASS-21	NR	≥10	21.0%	DASS-21	NR	≥8	27.8%	NA	NA	NA	NA
Oguntayo et al, 2022^81^	COVID-19 era	300	PHQ-9	0.86	≥10	33.7%	GAD-7	0.78	≥15	58.0%	IES-R	0.87	≥37	33.7%
Olashore et al, 2021^25^	COVID-19 era	373	NA	NA	NA	NA	Anxiety Rating Scale	0.96	≥25	13.5%	NA	NA	NA	NA
Olashore et al, 2022^82^	COVID-19 era	353	PHQ-9	NR	≥9	23.2%	Anxiety Rating Scale	NR	≥25	14.0%	NA	NA	NA	NA
Onchonga et al, 2021^83^	COVID-19 era	476	PHQ-9	NR	≥5, 10, 15; 20	35.1% mild; 33.8% moderate; 17.6% moderately severe; 13.4% severe	GAD-7	NR	≥5, 10, 15	53.6% mild; 26.9% moderate; 10.3% moderately severe; 9.2% severe	NA	NA	NA	NA
Osasona and Oderinde, 2023^54^	COVID-19 era	213	NA	NA	NA	NA	GAD-7	0.86	≥8	25.4%	NA	NA	NA	NA
Phiri et al, 2023^50^	COVID-19 era	435	SRQ	NR	≥8	28.1%	NA	NA	NA	NA	NA	NA	NA	NA
Quadri et al, 2021^26^	COVID-19 era	489	PHQ-2	NR	≥10	2.1% daily depressive symptoms pre-pandemic 20.0% during the pandemic	NA	NA	NA	NA	NA	NA	NA	NA
Sagaon-Teyssier et al, 2020^84^	COVID-19 era	135	PHQ-9	NR	≥10	18.5%	GAD-7	NR	≥7	21.5%	NA	NA	NA	NA
Shah et al, 2021^43^	COVID-19 era	433	PHQ-9	0.90	≥10	53.6%	GAD-7	0.91	≥7	44.3%	IES-R	0.97	≥26	31.0%
Shumye et al, 2022^6^	COVID-19 era	207	HAD	0.76	≥8	32.4%	NA	NA	NA	NA	PSS-SR	NR	≥13	26.5%
Siamisang et al, 2022^85^	COVID-19 era	447	DASS-42	0.95	≥10	21.0%	DASS-42	0.82	≥8	28.2%	NA	NA	NA	NA
Simbeza et al, 2023^51^	COVID-19 era	713	PHQ-9	NR	≥5	46.8%	NA	NA	NA	NA	NA	NA	NA	NA
Teshome et al, 2020^53^	COVID-19 era	798	NA	NA	NA	NA	GAD-7	0.81	≥6 ≥11	29.3% mild anxiety 6.3% moderate anxiety	NA	NA	NA	NA
Vancampfort et al, 2022^41^	COVID-19 era	108	NA	NA	NA	NA	NA	NA	NA	NA	PCL-5	NR	≥41	44.4%
Wayessa et al, 2021^48^	COVID-19 era	275	DASS-21	NR	NR	21.5%	NA	NA	NA	NA	NA	NA	NA	NA
Wayessa et al, 2023^55^	COVID-19 era	275	NA	NA	NA	NA	DASS-21	0.89	NR	25.5%	NA	NA	NA	NA
Workneh et al, 2023^44^	COVID-19 era	577	PHQ-9	0.84	≥10	2.3% at time 1, 6.5% at time 2	NA	NA	NA	NA	NA	NA	NA	NA
Yadeta et al, 2021^49^	COVID-19 era	265	PHQ-9	NR	≥10	14.2%	NA	NA	NA	NA	NA	NA	NA	NA
Yitayih et al, 2020^59^	COVID-19 era	249	NA	NA	NA	NA	NA	NA	NA	NA	IES-R	NR	≥9	78.3%

PC-PTSD (DSM-V), Primary Care – Posttraumatic Stress Disorder for Diagnostic Statistical Manual (DSM) V; PCL-C-PTSD, Checklist – Civilian Version; PCL-5-PTSD, Checklist for DSM-5; PSS, Perceived Stress Scale; STAI, State Trait Anxiety Scale; DTS, Davidson Trauma Scale; SARSQ, Stanford Acute Reaction Stress Questionnaire; PSS-SR, Symptom Scale for Posttraumatic Stress Disorder – Self Reporting Version; HARS, Hamilton Anxiety Rating Scale; DASS, Depression, Anxiety and Stress Scale; IES-R, Revised Impact of Event Scale; HAD, Hospital Depression and Anxiety Scale; PHQ, Patient Health Questionnaire; GAD, generalised anxiety disorder; BDI-II, Beck’s Depression Inventory-II; CAS, COVID-19 Anxiety Scale; SRQ, Self-Reporting Questionnaire; HSCL, Hopkin’s Symptoms Checklist; NR, not reported; NA, not assessed.

### Prevalence of depression, anxiety and PTSD among HCWs from SSA

All the included studies reported the prevalence of one or more of the mental disorders of interest in this review (depression, anxiety and PTSD). Across these studies, wide-ranging prevalence estimates of depression, anxiety or PTSD among HCWs from SSA were reported. Table 2 summarises the prevalence of each of these disorders as reported in these studies, including the measures used and the cut-off scores applied, where reported.

### Prevalence of depressive symptoms among HCWs from SSA

Fifty-two studies reported the prevalence of depressive symptoms, either exclusively or concurrently with other mental disorders. Across these studies, depressive symptoms were assessed using several measurement tools, including various versions of the Patient Health Questionnaire^86^ used in 29 studies,^4,23,24,26,27,29,31,32,37,42–45,47,49,51,65,66,68,70,72,75–78,81–84^ the Depression, Anxiety and Stress Scale^87^ used in 12 studies,^28,34,40,46,48,60,63,67,71,74,80,85^ Beck’s Depression Inventory^88^ used in 3 studies,^36,62,64^ the Hospital Anxiety and Depression Scale^89^ used in 5 studies,^6,61,69,73,79^ the Self-Reporting Questionnaire^90^ used in 2 studies^14,50^ and the Zung Self-Rating Depression Scale^91^ used in 1 study.^33^

Wide-ranging prevalence estimates of depressive symptoms were reported across studies (Table 2). Regardless of the measurement tool used, the prevalence of depression ranged from 2.1 to 75.7%. One study from Nigeria^27^ compared the prevalence of depressive symptoms between HCWs and the general population and reported a significantly higher prevalence among HCWs (35.1 versus 23.5%, *P* < 0.01). One study that was conducted across 13 countries compared the prevalence of daily depressive symptoms before and during the COVID-19 pandemic.^26^ In that study, the prevalence of daily depressive symptoms among 439 HCWs before and during the pandemic was 2.1 and 20.0%, respectively.

### Prevalence of anxiety symptoms among HCWs from SSA

Forty-five studies assessed the prevalence of anxiety symptoms, either exclusively or concurrently with other mental disorders. Across these studies, anxiety symptoms were assessed using several anxiety symptom screeners, including, the 7-item Generalised Anxiety Disorder Scale^92^ used in 22 studies,^27,30,32,37,42,43,45,47,52–54,65,68,70,72,75–78,81,83,84^ the Depression, Anxiety and Stress Scale^87^ used in 11 studies,^34,40,46,55,60,63,67,71,74,80,85^ the Anxiety Rating Scale^93^ used in 2 studies,^25,82^ the Hospital Anxiety and Depression Scale^89^ used in 4 studies,^61,69,73,79^ a 4-item version of the Patient Health Questionnaire^86^ used in 1 study,^23^ the State Trait Anxiety Inventory^94^ used in 1 study,^62^ the Coronavirus Anxiety Scale^95^ used in 2 studies,^38,76^ the Hopkins Symptom Checklist^96^ used in 1 study,^15^ the Hamilton Anxiety Rating Scale^97^ used in 1 study^7^ and the Death Anxiety Subscale of the Death Distress Scale^98^ used in 1 study.^32^

Across the 45 studies, wide-ranging prevalence estimates of anxiety symptoms, including symptoms of generalised anxiety disorder, trait anxiety, state anxiety, COVID-19 anxiety and death anxiety, were reported (Table 2). Overall, the prevalence of anxiety symptoms ranged from 4.8 to 96.5%. One study compared the symptoms of generalised anxiety disorder among 382 HCWs and 502 participants from the general population, and reported a prevalence of 58.4% and 49.6% among HCWs and the general population, respectively.^27^

### Prevalence of PTSD symptoms among HCWs from SSA

In total, 21 of the included studies reported the prevalence of PTSD symptoms among HCWs from SSA. Across these studies, PTSD symptoms were assessed using various PTSD symptom screeners, including the revised Impact of Event Scale^99^ used in 12 studies,^27,37,43,45,56,57,59,67,68,70,71,81^ the PTSD Checklist for Diagnostic Statistical Manual 5 (DSM-5)^100^ used in 3 studies,^15,41,58^ the PTSD Checklist – Civilian Version^101^ used in 2 studies,^35,39^ the Primary Care – Posttraumatic Stress Disorder for DSM-5^102^ used in 2 studies,^66,75^ the Stanford Acute Reaction Stress Questionnaire^103^ used in 1 study^64^ and the Symptom Scale for Posttraumatic Stress Disorder – Self-Reporting Version^104^ used in 1 study.^6^

Similar to depressive and anxiety symptoms, wide-ranging prevalence estimates of PTSD symptoms were also reported across studies (Table 2). The prevalence of PTSD symptoms among HCWs as reported across these studies ranged from 11.7 to 78.3%. One study from Nigeria compared the prevalence of PTSD symptoms among 382 HCWs and 502 participants from the general population, and reported a significantly higher prevalence among HCWs (52.6 versus 42.8%, *P* < 0.05).^27^

### Correlates of depression, anxiety and PTSD among HCWs from SSA

In total, 36 of the included studies reported the correlates of one or more of the mental disorders of interest in this review (depression, anxiety and PTSD). Tables 3, 4 and 5 present detailed summaries of these correlates, including their effect sizes. Across these studies, several correlates of depression, anxiety or PTSD among HCWs from SSA were reported. Overall, these correlates can be classified into sociodemographic, health-related, COVID-19-related and work-related correlates, and are reported as such in this paper.

**Table 3:** Correlates of depression among healthcare workers from sub-Saharan Africa

		Sociodemographic correlates		Health-related correlates		COVID-19-related correlates		Work-related correlates	
Author, year	Measure of effect (precision)	Risk indicators	Protective indicators	Risk indicators	Protective indicators	Risk indicators	Protective indicators	Risk indicators	Protective indicators
Ali et al, 2021^37^	AOR (95% CI)	NR	NR	NR	NR	Working at the front lines: 5.98 (1.31–27.33)	NR	NR	NR
Ayalew et al, 2021^63^	AOR (95% CI)	Female gender: 3.71 (2.31–5.97) Being married: 2.28 (1.34–3.86) Living alone: 1.87 (1.09–3.20)	NR	NR	NR	NR	NR	Being a nurse: 2.94 (1.44–5.99)	Working with in-patients: 0.53 (0.29–0.93)
Belete andAnbensaw, 2022^29^	AOR (95% CI)	Female gender: 1.94 (1.12–3.67) Being unmarried: 2.16 (1.12–4.15) Substance use: 2.67 (1.36–5.24)	NR	History of mental illness: 7.31 (2.27–23.49)	NR	NR	NR	NR	NR
Birhane et al, 2023^31^	AOR (95% CI)	NR	NR	Burnout: 11.88 (5.27–26.80)	NR	NR	NR	NR	NR
Falade et al, 2022^69^	AOR (95% CI)	Younger age: 1.51 (1.03–2.21)	Being a Christian: 0.50 (0.29–0.88)	NR	NR	Not satisfied with government support during the pandemic: 2.33 (1.45–3.45)	NR	NR	NR
GebreEyesus et al, 2021^70^	AOR (95% CI)	Male gender: 1.91 (1.04–3.50) Higher education level: 10.84 (3.31–35.48) Living with family: 5.82 (1.90–17.89)	NR	NR	NR	NR	NR	Being a pharmacist: 4.52 (1.88–11.01)	Being a physician: 0.20 (0.06–0.65)
Jemal et al, 2022^74^	AOR (95% CI)	Female gender: 2.01 (1.25–3.23)	NR	NR	NR	Working in COVID-19 treatment centre: 2.14 (1.05–4.39)	NR	Working in Oromiya region: 3.94 (1.94–8.09) Being a medical laboratory technologist: 4.69 (2.81–9.17)	NR
Kwobah et al, 2021^75^	AOR (95% CI)	Female gender: 1.47 (1.09–2.00) Being unmarried: 1.47 (1.07–2.01)	NR	NR	NR	NR	NR	NR	Having more years of experience: 0.50 (0.28–0.88)
Mbanga et al, 2019^4^	AOR (95% CI)	NR	NR	Burnout: 1.21 (1.08–1.35)	NR	NR	NR	Greater number of night shifts per week: 1.58 (1.01–2.48)	NR
Mc Magh et al, 2023^76^	AOR (95% CI)	Female gender: 4.86 (1.08–21.90)	NR	NR	NR	NR	NR	NR	NR
Mekonen et al, 2021^40^	AOR (95% CI)	NR	NR	History of mental illness: 6.57 (1.66–26.01) Having chronic illness: 4.48 (1.86–10.82)	NR	Not having guidelines on COVID-19 management: 2.26 (1.21–4.21) Negative feedback from family and friends about being at the front lines: 2.19 (1.27–3.79)	NR	NR	NR
Mulatu et al, 2021^78^	AOR (95% CI)	Being unmarried: 2.50 (1.40–4.60) Lower education: 2.10 (1.40–6.20)	NR	NR	NR	Working at the front lines: 2.40 (1.20–2.50)	NR	NR	NR
Naidoo et al, 2020^32^	AOR (95% CI)	Older age: 21.23 (1.64–275.20) Female gender: 3.85 (1.21–12.22)	NR	Burnout: 13.83 (3.08–62.00)	NR	NR	NR	Being a non-specialist doctor: 6.45 (1.12–37.09)	NR
Olashore et al, 2022^82^	*β* (*t*)	NR	Older age: −0.10 (−2.08)	Neuroticism: 0.22 (4.45)	Resilience: −0.35 (−7.22)	NR	NR	NR	NR
Phiri et al, 2023^50^	AOR (95% CI)	NR	NR	Burnout: 3.20 (1.90–5.40)	NR	Expecting to be infected with COVID-19 within the next 12 months: 2.70 (1.30–5.50) Previously infected with COVID-19: 2.58 (1.40–5.00)	NR	NR	NR
Segaon-Teyssier et al, 2020^84^	IRR (95% CI)	Female gender: 1.60 (1.16–2.21) Higher number of economically dependent family members: 2.48 (1.31–4.68)	NR	NR	NR	NR	Working in centres with face masks: 0.49 (0.34–0.70)	Higher perception of moral transgression in the workplace: 1.04 (1.02–1.05) Working in centres offering consultations to the general population: 4.67 (1.79–12.20)	Being a nurse: 0.40 (0.20–0.77) Higher nurse:patient ratio: 0.09 (0.01–0.57)
Shah et al, 2021^43^	AOR (95% CI)	Female gender: 1.95 (1.12–3.42)	NR	NR	NR	Working at the front lines: 3.55 (1.77–7.11)	NR	Being a doctor: 2.24 (1.27–3.94)	NR
Siamisang et al, 2022^85^	AOR (95% CI)	Smoking: 2.39 (1.23–4.67)	NR	NR	NR	Experience of stigma due to working at the front lines: 1.68 (1.01–2.81)	Working at the largest COVID-19 isolation centre: 0.25 (0.15–0.43)	NR	NR
Wayessa et al, 2021^48^	AOR (95% CI)	Younger age: 2.35 (1.13–3.95) Large family size: 3.56 (1.09–11.62) Alcohol use: 4.31 (1.76–10.55)	NR	Having medical illness: 9.56 (3.71–24.59)	NR	Lack of knowledge on COVID-19: 15.34 (6.32–37.21)	Having training on COVID-19: 0.37 (0.17–0.81)	NR	NR
Workneh et al, 2023^44^	AOR (95% CI)	Female gender: 3.96 (1.08–14.51)	NR	NR	NR	Positive COVID-19 result: 7.25 (1.32–39.40) Lack of COVID-19 guidelines: 3.22 (1.11–9.35)	NR	NR	NR
Yadeta et al, 2021^49^	AOR (95% CI)	Female gender: 2.34 (1.09–5.02)	NR	NR	NR	Perceived susceptibility to COVID-19: 4.05 (1.12–14.53)	NR	NR	NR

*β*, beta coefficient (adjusted); AOR, adjusted odds ratio; IRR, incidence rate ratio; NR, none reported.

**Table 4:** Correlates of anxiety among healthcare workers from sub-Saharan Africa

		Sociodemographic correlates		Health-related correlates		COVID-19-related correlates		Work-related correlates	
Author, year	Measure of effect (precision)	Risk indicators	Protective indicators	Risk indicators	Protective indicators	Risk indicators	Protective indicators	Risk indicators	Protective indicators
Ayalew et al, 2021^63^	AOR (95% CI)	Older age: 3.15 (1.04–6.56) Female gender: 3.25 (2.01–5.25) Being married: 1.69 (1.01–2.87)	NR	NR	NR	NR	NR	Being a nurse: 3.32 (1.63–6.78)	NR
Belayneh et al, 2021^7^	AOR (95% CI)	Cigarette smoking: 2.48 (1.18–5.18)	NR	NR	NR	NR	NR	Work overload: 1.35 (1.16–3.76) Working night shifts: 2.41 (1.19–6.87)	NR
Cenat et al, 2022^15^	*β* (95% CI)	NR	Social support: −0.14 (−0.17 to −0.11)	EVD exposure: 0.12 (−0.01 to 0.25) EVD stigma: 0.07 (0.02–0.11)	NR	NR	NR	NR	NR
Dawood et al, 2021^67^	*β* (s.e.)	NR	Male gender: −3.69 (1.39)	NR	NR	NR	NR	NR	NR
Falade et al, 2022^69^	AOR (95% CI)	NR	NR	NR	History of mental illness: 0.23 (0.05–0.94)	NR	NR	Being a health worker: 4.08 (1.20–8.35)	
GebreEyesus et al, 2021^70^	AOR (95% CI)	Older age: 7.98 (1.44–44.17) Higher education level: 3.24 (1.00–10.48) Lower income: 1.87 (1.14–3.06)	NR	NR	NR	Having infected family members: 3.30 (1.50–7.23)	NR	NR	NR
Jemal et al, 2022^74^	AOR (95% CI)	Female gender: 1.91 (1.27–2.86)	NR	NR	NR	Working in COVID-19 treatment centre: 3.49 (2.24–6.97)	NR	Working in Oromiya region: 1.85 (1.14–2.99) Being a medical laboratory technologist: 2.75 (1.78–4.79)	NR
Kibret et al, 2020^52^	AOR (95% CI)	Older age: 3.05 (1.70–5.47) Being married: 3.56 (2.30–6.38)	NR	Having a chronic illness: 3.43 (1.59–7.43)	NR	Having a family member with suspected COVID-19: 5.20 (2.11–12.78) Not having access to PPE: 2.55 (1.43–4.56)	NR	NR	NR
Kwobah et al, 2021^75^	AOR (95% CI)	Female gender: 1.72 (1.25–2.37) Being unmarried: 1.41 (1.02–1.96)	NR	NR	NR	NR	NR	Working in a private health facility: 1.57 (1.13–2.20)	Having more years of experience: 0.49 (0.27–0.97)
Mc Magh et al, 2023^76^	AOR (95% CI)	Female gender: 2.78 (1.39–5.57) Non-African: 2.54 (1.00–6.42)	NR	NR	NR	NR	NR	NR	NR
Mekonen et al, 2021^40^	AOR (95% CI)	NR	NR	Having chronic illness: 3.78 (1.25–11.49)	NR	Not having guidelines on COVID-19 management: 3.12 (1.69–5.76) Fear of infecting family: 2.34 (1.29–4.23)	NR	NR	NR
Mulatu et al, 2021^78^	AOR (95% CI)	Lower income: 8.10 (2.50–30.40)	NR	NR	NR	Working at the front lines: 2.10 (1.10–3.90)	NR	NR	NR
Muliira et al, 2015^8^	*β* (s.e.)	NR	NR	Coping with maternal death using planning: 2.19 (0.58) Coping with maternal death using active coping: 1.23 (0.56) Coping with maternal death using acceptance: 1.10 (0.59)	NR	NR	NR	Witnessed two or more maternal deaths in the past 2 years: 1.16 (0.15) Being in charge of 4 or more maternal deaths: 1.64 (0.62) Lack of professional training in handling death situations: 1.20 (0.37)	
Naidoo et al, 2020^32^	AOR (95% CI)	Older age: 27.62 (1.04–732.21)	NR	Burnout: 8.62 (2.21–33.59)	NR	NR	NR	NR	NR
Olashore et al, 2021^25^	*β* (*t*)	NR	NR	Neuroticism: 0.51 (10.59)	Resilience: −0.34 (−7.11) Social support: −0.08 (−2.11)	NR	NR	NR	NR
Olashore et al, 2022^82^	*β* (*t*)	NR	Low education status: −0.13 (−2.73)	Neuroticism: 0.31 (6.50)	Resilience: −0.31 (−6.40)	NR	NR	NR	NR
Segaon-Teyssier et al, 2020^84^	IRR (95% CI)	NR	NR	Perceived lower health status: 1.52 (1.05–2.22)	NR	NR	Working in centres with face masks: 0.38 (0.21–0.67)	Higher perception of moral transgression in the workplace: 1.03 (1.01–1.04)	Higher nurse:patient ratio: 0.05 (0.01–0.62)
Shah et al, 2021^43^	AOR (95% CI)	Female gender: 1.95 (1.04–3.65)	NR	NR	NR	Working at the front line: 2.51 (1.20–5.25)	NR	Being a doctor: 2.29 (1.21–4.32)	NR
Siamisang et al, 2022^85^	AOR (95% CI)	Tertiary education: 1.82 (1.07–3.07) Having household members with chronic heart/lung disease: 2.05 (1.20–3.50)	NR	NR	NR	Losing relatives or friends to COVID-19: 1.72 (1.10–2.70)	Working at the largest COVID-19 isolation centre: 0.49 (0.31–0.77)	NR	NR
Teshome et al, 2020^53^	APOR (95% CI)	NR	NR	No confidence in coping with stress: 4.81 (1.63–4.61)	Not feeling overwhelmed by the demands of everyday life: 0.52 (0.37–0.73) Not feeling that you cannot make it: 0.44 (0.31–0.63)	Contact with suspected or confirmed case: 1.97 (1.29–3.12) Lack of COVID-19 updates: 4.81 (2.96–7.84) COVID-related worry: 1.85 (1.12–3.06)	NR	NR	NR
Weyessa et al, 2023^55^	AOR (95% CI)	Younger age: 5.39 (1.35–21.54) Drug use: 1.52 (1.35–11.21) Alcohol use: 3.85 (1.94–7.65) Lower monthly income: 12.58 (2.13–74.38)	NR	Medical illness: 8.74 (3.61–21.18)	NR	NR	NR	NR	NR

*β*, beta coefficient (adjusted); AOR, adjusted odds ratio; APOR, adjusted proportional odds ratio; IRR, incidence rate ratio; PTSD, post-traumatic stress disorder; EVD, Ebola virus disease; PPE, personal protective equipment; NR, none reported.

**Table 5:** Correlates of post-traumatic stress disorder among healthcare workers from sub-Saharan Africa

		Sociodemographic correlates		Health-related correlates		COVID-19-related correlates		Work-related correlates	
Author, year	Measure of effect (precision)	Risk indicators	Protective indicators	Risk indicators	Protective indicators	Risk indicators	Protective indicators	Risk indicators	Protective indicators
Ayalew et al, 2022^57^	AOR (95% CI)	Female gender: 1.91 (1.19–3.05) Being married: 1.87 (1.12–3.14)	NR	NR	NR	NR	NR	Being a nurse: 3.31 (1.66–6.63)	Working with in-patients: 0.43 (0.24–0.75) Working in units apart from the emergency unit: 0.49 (0.25–0.96) Working in the out-patient department: 0.48 (0.24–0.97)
Asnakew et al, (2021)^56^	AOR (95% CI)	Older age: 3.95 (1.74–8.98) Poor social support: 4.41 (2.65–7.30)	NR	Perceived health worker-related stigma: 1.97 (1.01–3.85) History of mental illness: 8.08 (2.18–29.98) Having medical illness: 4.65 (1.65–13.12)	NR	Lack of PPE: 2.57 (1.37–4.85)	NR	NR	Being a physician: 0.15 (0.04–0.56)
Cenat et al, 2022^15^	*β* (95% CI)	NR	Social support: −0.30 (−0.38 to −0.22)	EVD exposure: 0.53 (0.21–0.86) EVD stigma: 0.14 (0.03–0.26)	NR	COVID-19 stigma: 0.22 (0.10–0.33)	NR	NR	NR
Dawood et al, 2021^67^	*β* (s.e.)	NR	Male gender: −5.60 (2.28)	NR	NR	Perceiving COVID-19 as a risk: 9.05 (3.27)	Pandemic preparedness: −5.53 (2.26)	NR	NR
Hajure et al, 2021^71^	AOR (95% CI)	Tobacco use: 6.76 (2.15–21.20) Alcohol use: 6.28 (2.03–19.50) Khat use: 5.74 (1.83–18.10)	NR	Depression: 10.54 (2.87–38.70)	NR	NR	NR	NR	NR
Kabunga and Okalo, 2021^39^	AOR (95% CI)	NR	Social support: 0.49 (0.34–0.60)	NR	NR	Fear of getting infected with COVID-19: 3.10 (2.17–4.43)	NR	Increased workload: 1.65 (1.16–2.34)	NR
Mulatu et al, 2021^78^	AOR (95% CI)	NR	NR	NR	NR	Working at the front lines: 5.90 (2.20–15.90)	NR	NR	NR
Olashore et al, 2018^35^	AOR (95% CI)		NR	Neuroticism: 2.72 (1.19–6.24)	NR	NR	NR	Exposure to workplace violence in the last 12 months: 3.26 (1.49–7.16)	NR
Shah et al, 2021^43^	AOR (95% CI)	Female gender: 2.18 (1.32–3.60)	NR	NR	NR	Working at the front lines: 1.89 (1.10–3.17)	NR	NR	NR
Shumye et al, 2022^6^	AOR (95% CI)	Female gender: 3.20 (1.62–6.36) Substance use: 2.11 (1.21–3.69)	NR	Poor sleep quality: 3.68 (1.93–7.02)	NR	Working in high-risk COVID-19 wards: 2.84 (1.49–5.41	NR	NR	NR
Yitayih et al, 2020^59^	AOR (95% CI)	Younger age: 24.50 (1.20–492.60)	NR	Insomnia: 19.20 (6.00–61.50)	NR	Not having daily updates on COVID-19: 2.60 (1.00–6.60)	NR	Stigmatisation and rejection in the neighbourhood because of hospital work: 2.70 (1.10–6.40)	NR

*β*, beta coefficient (adjusted); AOR, adjusted odds ratio; EVD, Ebola virus disease; PPE, personal protective equipment; NR, none reported.

### Correlates of depressive symptoms among HCWs from SSA

Twenty-one of the included studies^4,29,31,32,40,43–45,48–50,63,69,70,74–76,78,82,84,85^ reported the correlates of depressive symptoms among HCWs (Table 3). The sociodemographic factors that were reported to significantly increase the risk of depressive symptoms among HCWs include older age,^32^ younger age,^48,69^ female gender,^29,32,43,44,49,63,74–76,84^ being unmarried,^29,75,78^ being married,^63^ living alone,^63^ male gender,^70^ higher education,^70^ lower education,^78^ living with family,^70^ having a higher number of economically dependent family members,^84^ being a Christian^69^ and substance use.^29,85^ Older age was the only sociodemographic factor reported to be protective against depressive symptoms, reported in only one study.^82^

Among health-related factors reported to increase the risk of depressive symptoms were having a chronic medical illness,^40,48^ history of mental illness,^29,40^ neuroticism^82^ and burnout.^4,31,32,50^ Resilience was the only health-related factor reported to lower the risk of depressive symptoms among HCWs, reported in one study.^82^

Several COVID-19-related factors were reported to increase the risk of depressive symptoms among HCWs, including working on the front lines,^37,43,78^ being infected with COVID-19,^44,50^ expecting to be infected with COVID-19 within the next 12 months,^50^ higher perceived susceptibility to COVID-19,^49^ lack of knowledge on COVID-19,^48^ not having guidelines on COVID-19 management,^40,44^ negative feedback from family and friends regarding being on the front lineS,^40^ not satisfied with government support during the pandemic,^69^ working in a COVID-19 treatment centre^74^ and experiencing stigma due to working on the front lines.^85^ Having training in COVID-19 management,^48^ having personal protective equipment^84^ and working in the largest COVID-19 isolation centre^85^ were the only COVID-19-related factors reported to be protective against depressive symptoms among HCWs during the pandemic.

Among work-related factors reported to increase the risk of depressive symptoms were having greater numbers of night shifts per week,^4^ being a nurse,^63^ being a pharmacist,^70^ being a physician,^70^ being a doctor,^43^ being a medical laboratory technologist,^74^ having a greater perception of moral transgression in the workplace,^84^ working in centres offering consultations to the general population as opposed to only people living with HIV^84^ and working outside the capital city.^74^ Only four work-related factors were reported to be protective against depressive symptoms, namely having more years of experience,^75^ being a nurse as opposed to other cadres of HCWs,^84^ a higher nurse:patient ratio^84^ and working in an in-patient department.^63^

### Correlates of anxiety symptoms among HCWs from SSA

In total, 21 of the included studies^7,8,15,25,32,40,43,52,53,55,63,67,69,70,74–76,78,82,84,85^ reported the correlates of anxiety symptoms among HCWs from SSA (Table 4). Across these studies, the sociodemographic factors that were reported to increase the risk of anxiety symptoms include older age,^32,52,63,70^ younger age,^55^ being married,^52,63^ female gender,^43,63,74–76^ being unmarried,^75^ male gender,^67^ higher education,^70,85^ lower income,^55,70,78^ having household members with chronic heart or lung disease,^85^ being of non-African descent^76^ and alcohol or other drug use.^7,55^ On the other hand, low education^82^ and social support^15,25^ were the only reported protective factors of anxiety symptoms.

The health-related factors reported to be associated with a higher risk of anxiety symptoms were having a chronic illness,^40,52,55^ neuroticism,^25,82^ burnout,^32^ having a history of mental illness,^69^ exposure to Ebola virus disease,^15^ experiencing Ebola virus disease stigma,^15^ coping with maternal death using planning, active coping or acceptance as coping strategies,^8^ lack of confidence in coping with stress^53^ and having a lower perceived health status.^84^ Resilience,^25,82^ not feeling overwhelmed by the demands of everyday life^53^ and not feeling that you cannot make it^53^ were the health-related factors reported to lower the risk of anxiety symptoms.

The COVID-19-related factors reported to increase the risk of anxiety symptoms include having a family member with suspected COVID-19,^52^ lack of access to personal protective equipment,^52^ lack of COVID-19 management guidelines,^40^ having infected family members,^70^ working on the front lines,^43,78^ working in a COVID-19 treatment centre,^74^ losing relatives or friends to COVID-19,^85^ contact with suspected or confirmed case of COVID-19,^53^ lack of COVID-19 updates^53^ and COVID-19-related worry.^53^ Working in the largest COVID-19 isolation centre^85^ and having personal protective equipment^84^ were the only COVID-19-related factors reported to be protective against anxiety symptoms during the pandemic.

Among work-related factors reported to increase the risk of anxiety symptoms among HCWs were working in a private facility,^75^ being a nurse,^63^ being a doctor,^43^ being a health worker,^69^ working outside the capital city,^74^ being a medical laboratory technologist,^74^ work overload,^7^ working night shifts,^7^ witnessing two or more maternal deaths in the past 2 years,^8^ being in charge of four or more maternal deaths,^8^ having a greater perception of moral transgression in the workplace^84^ and lack of training in handling death situations.^8^ Having more years of experience^75^ and a higher nurse:patient ratio^84^ were the only reported protective factors against anxiety symptoms.

### Correlates of PTSD symptoms among HCWs from SSA

Eleven of the included studies^6,15,35,39,43,56,57,59,67,71,78^ reported the correlates of PTSD symptoms among HCWs from SSA (Table 5). Across these studies, the reported sociodemographic risk factors of PTSD symptoms include female gender,^6,43,57^ being married,^57^ male gender,^67^ substance use,^6,71^ older age,^56^ younger age^59^ and poor social support.^56^ Higher social support was the only reported sociodemographic protective factor of PTSD symptoms.^15,39^

The health-related factors that were reported to increase the risk of PTSD symptoms include having a chronic illness,^56^ neuroticism,^35^ insomnia,^59^ depression,^71^ exposure to Ebola virus disease,^15^ experience of Ebola virus disease stigma,^15^ poor sleep quality,^6^ experiencing stigma associated with being a health worker^56,59^ and history of mental illness.^56^ No study reported any protective health-related factors of PTSD symptoms.

The reported COVID-19-related risk factors of PTSD symptoms include fear of getting infected,^39^ perceiving COVID-19 as a risk,^67^ working on the front lines,^43,78^ COVID-19-related stigma,^15^ working in high-risk COVID-19 wards,^6^ not having daily updates on COVID-19^59^ and lack of personal protective equipment.^56^ Pandemic preparedness was the only reported protective factor against PTSD symptoms among HCWs.^67^

The only work-related factors reported to increase the risk of PTSD symptoms include being a nurse,^57^ experiencing workplace violence^35^ and increased workload.^39^ Working with in-patients,^57^ working in units other than the emergency department,^57^ working in the out-patient department^57^ and being a physician^56^ were the only reported protective factors against PTSD symptoms.

### Impact of the COVID-19 pandemic on depression, anxiety and PTSD among HCWs from SSA

From this review, it appears that the COVID-19 pandemic increased the burden and/or risk of depression, anxiety and PTSD among HCWs in SSA. As earlier reported, 55 of the included studies were conducted during the pandemic while the remaining 14^7,8,14,24,28–36^ were conducted prior to the onset of the pandemic. Comparing the results from these studies, those conducted during the pandemic appear to show relatively higher prevalence estimates of depression, anxiety and PTSD compared with those conducted before the pandemic. Wide-ranging prevalence estimates were reported for these mental disorders both during and before the pandemic. The prevalence of depression ranged from 2.1 to 62.2% pre-pandemic and 6.5 to 75.7% during the pandemic. Similarly, the prevalence of anxiety ranged from 19.8 to 46.6% pre-pandemic while it ranged from 4.8 to 96.5% in the later phases. The prevalence of PTSD pre-pandemic was 18.4% (only 1 study assessed PTSD pre-pandemic). The prevalence of PTSD ranged from 11.7 to 78.3% during the pandemic.

Despite the wide ranges observed, the reported prevalence of depressive symptoms was as high as 75.7% during the pandemic^74^ compared with 62.2% before.^4^ When comparing the prevalence of depressive symptoms among the same set of HCWs before and during the pandemic, 1 study^26^ reported a higher prevalence during the pandemic (20.0% during the pandemic versus 2.1% pre-pandemic). The prevalence of anxiety symptoms was as high as 96.5% during the pandemic^69^ compared with 46.6% before.^8^ Finally, the prevalence of PTSD symptoms was as high as 78.3% during the pandemic^75^ compared with 18.4% before.^35^ Regarding COVID-19 increasing the risk of these disorders, many COVID-19-related factors were reported to increase the risk of depression, anxiety and PTSD among HCWs from SSA, with some of these increasing the risk of these disorders by up to nine times (please see the subtopics on the correlates of each disorder and Tables 3, 4 and 5).

### Quality of the included studies

The quality scores of the included studies are presented in Supplementary File 3. Overall, 32 of the included studies were rated to be of good quality, 31 of satisfactory quality and 6 of unsatisfactory quality. Based on a cut-off score of ≥7 on the NOS, only 32 of these studies were of high quality while the remaining 37 were of low quality and with a high risk of bias. When comparing the results of high- and low-quality studies, the prevalence of depression as reported by the former ranged from 6.7 to 75.7%, compared with a range of 2.1 to 66.5% by the latter. The prevalence of anxiety as reported by the high-quality studies ranged from 3.2 to 79.0%, compared with 4.8 to 96.5% by the low-quality studies. The prevalence of PTSD as reported by the high-quality studies ranged from 12.0 to 61.7%, compared with 11.7 to 40.2% reported by the low-quality studies.

## Discussion

We conducted this systematic review to summarise the available evidence on the burden and factors associated with depression, anxiety and PTSD among HCWs from SSA. A total of 69 studies met our inclusion criteria and were reviewed. Almost 80% of the included studies (*n* = 55) specifically investigated the burden of depression, anxiety and/or PTSD, and their associated factors, among HCWs in the context of the COVID-19 pandemic. Given the virus’s high rates of transmission and associated mortality, this might highlight the rapid and important response by researchers to understanding the mental health of a key group of the population that was at the forefront of the fight against the pandemic.

In this review, we note that most of the included studies focused on either depression (*n* = 52) or anxiety (*n* = 45), with a limited focus on PTSD (*n* = 21). In part, this may be attributed to the paucity of tools adequately validated for PTSD in this region. Evidence from a systematic review that assessed the availability of validated screening tools for common mental disorders in low- and middle-income countries found that, of the 275 identified validations, only 13 were for PTSD measurement tools compared with 175 for measures of depression and 24 for measures of anxiety.^105^ Further studies aimed at developing and/or validating the measures of PTSD among HCWs in this setting are needed.

Wide-ranging prevalence estimates of depression, anxiety and PTSD among HCWs from SSA were reported across the included studies in this review; the prevalence of depressive, anxiety and PTSD symptoms ranged from 2.1 to 75.7, 4.8 to 96.5 and 11.7 to 78.3%, respectively. Despite the wide ranges and the high estimates observed, these prevalence estimates should be interpreted with caution because of several factors that may have resulted in biased estimates. Many of the participants in the included studies were recruited non-randomly, information on the reliability and validity of the measures used in these studies was sparse and most of the included studies were of poor quality. With that said, wide-ranging prevalence estimates of these disorders were also reported in a recent narrative review involving HCWs from SSA during the COVID-19 pandemic.^17^ Similar wide-ranging findings have also been reported in reviews involving HCWs from across the globe, although these reviews also focused on the COVID-19 pandemic.^106,107^ Differences in sample sizes, study populations, measurement tools, cut-off scores for similar measures and study settings (for example, exposure to different risk factors or differences in exposure levels for similar risk factors) may explain the wide variation in the reported prevalence estimates of depression, anxiety and PTSD among HCWs from SSA. Despite the wide-ranging estimates, the prevalence of these disorders among HCWs from this region appears to be high, even when compared with that in the general population as reported in one of the included studies.^27^ Studies from other regions that recruited a comparison group from the general population also report higher estimates among HCWs.^108^ However, given that only one study in this review included a comparison group, future studies on the mental health of HCWs should aim to include comparison groups to enable more definite conclusions. That said, the high burden noted here calls for targeted psychosocial interventions to support this key and important group. This is especially important because the existence of these disorders among HCWs has been associated with negative clinical implications such as poor-quality care and medical errors.^1,2^

Several sociodemographic, health-related, COVID-19-related and work-related correlates of depression, anxiety and PTSD were identified in this review, but are largely limited to one study, making it difficult to draw any conclusions. Nevertheless, there seemed to be consensus in more than one study regarding female gender, younger age, being unmarried, burnout, having a chronic illness, having a history of mental illness, substance use and working at the front line during the COVID-19 pandemic as significant correlates of depressive symptoms among HCWs from SSA. Similarly, for anxiety symptoms, older age, female gender, being married, higher education, lower income, having a chronic illness, neuroticism, resilience, social support and working at the front line during the pandemic were the only correlates with consensus across studies. Female gender, substance use, depression, working at the front line during the pandemic and social support were the only correlates of PTSD with consensus across studies. Many of these factors have also been reported in other reviews.^17,106,107,109,110^ However, since most of these reviews focused on studies conducted during the pandemic, most of the identified factors were COVID-19 related. Empirical studies from outside SSA that were conducted prior to the pandemic also report most of these factors.^111–116^

From this review, it appears that the COVID-19 pandemic increased the burden of depression, anxiety and PTSD among HCWs from SSA. Although almost all the included studies did not directly compare the burden of these disorders before and during the pandemic, when comparing the evidence from studies conducted before and during the pandemic the reported prevalence estimates of these disorders across studies appear to be higher during the pandemic compared with before. Evidence from the only study that compared the burden of these, specifically depressive symptoms, among the same set of HCWs before and during the pandemic also shows a significantly higher burden during the pandemic.^26^ The pandemic also increased the risk of these disorders in this population, with many COVID-19-related risk factors of depression, anxiety and PTSD being identified in this review. Pandemics and other health emergencies have been known to negatively impact the mental health of HCWs.^11,12,18^ However, in this review, all the evidence is from cross-sectional studies. In the event of similar health emergencies in the future, longitudinal studies are needed not only to explore the immediate impact of such emergencies on healthcare workers’ mental health but also the long-term effects.

Overall, this review shows that a high proportion of HCWs from SSA experience significant symptoms of depression, anxiety and PTSD. These symptoms appear to have been elevated by the COVID-19 pandemic and are associated with several sociodemographic, health-related, COVID-19-related and work-related factors. Targeted interventions are needed to address the burden of common mental disorders in this population.

### Strengths and limitations of this review

Our database search included African-based databases, including the African Index Medicus and African Journals Online. This allowed for the inclusion of relevant articles that would potentially have been missed had we focused on only Western-based databases. Additionally, our review was not restricted to a specific time period such as the pandemic, allowing us to provide a comprehensive picture of the mental health of HCWs from this region while also highlighting the potential impact of the COVID-19 pandemic. This review also has certain limitations worth highlighting. Because we restricted our search to only those studies published in English, we might have missed out on other relevant studies published in other languages. Additionally, all the included studies were cross-sectional in design, limiting our conclusions about causality and temporal associations. The limited information regarding the reliability and validity of the measurement tools used in most studies, as well as the non-random recruitment of study participants, may have introduced bias that undermines the accuracy, conclusions and generalisability of study findings.

### Implications of the results for practice, policy and future research

Despite the limitations, this study has implications for practice, policy and future research. The high burden of depression, anxiety and PTSD among HCWs from SSA calls for an urgent need for policies and interventions to address this burden in this key population. Such interventions should be multi-component in nature, given the multifactorial nature of the correlates of these disorders. The high burden also calls for the integration of mental health screening and treatment into the available healthcare package for HCWs in SSA. All the included studies were cross-sectional in design. There is a need for future research to focus on longitudinal designs to map the longitudinal trends of mental health among HCWs. Future research could also utilise different study designs to explore causality and confidently ascertain the reported associations. The heterogeneity of the outcome measures used precluded a meta-analysis. We recommend that future studies use standardised measures to allow for accurate and meaningful comparisons. Finally, since very few of the studies had recruited comparison groups, future studies on the mental health of HCWs should aim to include comparison groups to enable more definite conclusions.

## Supporting information

Too et al. supplementary material 1 Too et al. supplementary material

Too et al. supplementary material 2 Too et al. supplementary material

Too et al. supplementary material 3 Too et al. supplementary material

## Data Availability

The data supporting the conclusions presented in this article are available within this article and its supplementary material.

## Supplementary material

The supplementary material is available online at https://doi.org/10.1192/bjo.2025.10818
