# Supplementary material for: Prevalence and factors associated with depression, anxiety and post-traumatic stress disorder among healthcare workers from sub-Saharan Africa: systematic review
Source: BJPsych Open. 2025 Sep 8;11(5):e192. doi: 10.1192/bjo.2025.10818 (PMC12451537; doi:10.1192/bjo.2025.10818)
Supplement: Too et al. supplementary material 1 — Too et al. supplementary material [file S2056472425108181sup001.docx]

**("Depression" [MeSH Terms] OR "Depressive Disorder" [MeSH Terms] OR "Depressive Disorder, Major" [MeSH Terms] OR Depress* [All Fields] OR "Depressive symptoms" [All Fields] OR "Symptoms of depression" [All Fields] OR "Mental illness" [All Fields] OR "Mental health" [MeSH Terms] OR "Anxiety" [MeSH Terms] OR "Anxiety Disorders" [MeSH Terms] OR Anxi* [All Fields] OR "Anxiety Symptoms" [All Fields] OR "Symptoms of anxiety" [All Fields]) OR "Post traumatic stress disorder" [All Fields] OR PTSD [MeSH Terms] OR "Post-traumatic stress disorder" [All Fields] OR "post-traumatic stress" [All Fields] OR "post-traumatic stress syndrome" [All Fields] OR "delayed stress syndrome" [all Fields] OR "delayed stress disorder" [All Fields] OR "PTSS" [All Fields]) AND ("healthcare workers" [All Fields] OR nurs* [All Fields] OR "physicians" [All Fields] OR "psychologists" [All Fields] OR "doctors" [All Fields] OR "social workers" [All Fields] OR "community health workers" [All Fields] OR "health personnel" [All Fields] OR midwi* [All Fields] OR "medical officers" [All Fields] OR "clinical officers" [All Fields] OR "health workers" [All Fields]) AND ("Africa South of the Sahara"[tiab] OR "Sub Saharan Africa"[tiab] OR "sub-Saharan Africa"[tiab] OR "Sub-Saharan Africa"[tiab] OR "Angola"[tiab] OR "Benin"[tiab] OR "Botswana"[tiab] OR "Burkina Faso"[tiab] OR "Upper Volta"[tiab] OR "Burundi"[tiab] OR "Cameroon"[tiab] OR "Cape Verde"[tiab] OR "Central African Republic"[tiab] OR "Chad"[tiab] OR "Comoros" [tiab] OR "Congo"[tiab] OR "Cote D'ivoire"[tiab] OR "Ivory Coast"[tiab] OR "Zaire"[tiab] OR "Democratic Republic Of The Congo"[tiab] OR " French Somaliland"[tiab] OR "Djibouti"[tiab] OR "Equatorial Guinea"[tiab] OR "Eritrea"[tiab] OR "Ethiopia"[tiab] OR "Gabonese Republic"[tiab] OR "Gabon"[tiab] OR "Gambia"[tiab] OR "Gold Coast"[tiab] OR "Ghana"[tiab] OR "Guinea"[tiab] OR "Guinea-Bissau"[tiab] OR "Kenya"[tiab] OR "Basutoland"[tiab] OR "Lesotho"[tiab] OR "Liberia"[tiab] OR "Malagasy Republic"[tiab] OR "Madagascar"[tiab] OR "Nyasaland"[tiab] OR "Malawi"[tiab] OR "Mali"[tiab] OR "Mauritania"[tiab] OR "Mauritius"[tiab] OR "Mayotte"[tiab] OR "Mozambique"[tiab] OR "Namibia"[tiab] OR "Niger"[tiab] OR "Nigeria"[tiab] OR "Reunion"[tiab] OR "Rwanda"[tiab] OR "Ruanda-Urundi"[tiab] OR "Sao Tome & Principe"[tiab] OR "Sao Tome"[tiab] OR "Senegal"[tiab] OR "Seychelles"[tiab] OR "Sierra Leone"[tiab] OR "Somalia"[tiab] OR "South Africa"[tiab] OR "South Sudan"[tiab] OR "Sudan"[tiab] OR "Swaziland"[tiab] OR "Eswatini"[tiab] OR "Togolese Republic"[tiab] OR "Togo"[tiab] OR "Uganda"[tiab] OR "United Republic Of Tanzania"[tiab] OR "Tanzania"[tiab] OR "Zambia"[tiab] OR "Zimbabwe"[tiab] OR "Rhodesia"[tiab] OR "Africa Eastern"[tiab] OR "Africa Southern"[tiab] OR "Africa Western"[tiab] OR "Africa Central" [tiab]) NOT review [Title/Abstract] NOT editorial [Title/Abstract] NOT letter [Title/Abstract] NOT comment [Title/Abstract] NOT case studies [Title/Abstract] NOT case series [Title/Abstract]**
