# Supplementary material for: Prevalence and factors associated with depression, anxiety and post-traumatic stress disorder among healthcare workers from sub-Saharan Africa: systematic review
Source: BJPsych Open. 2025 Sep 8;11(5):e192. doi: 10.1192/bjo.2025.10818 (PMC12451537; doi:10.1192/bjo.2025.10818)
Supplement: Too et al. supplementary material 2 — Too et al. supplementary material [file S2056472425108181sup002.docx]

Supplementary File 2: Characteristics of the included studies

| **Author (year)** | **Country** | **Study design** | **Cadre** | **Study setting** | **Date of data collection** | **Mode of data collection** | **Sampling method** | **Sample size** | **Mental Health Outcomes** | **Reported study limitations** |
| --- | --- | --- | --- | --- | --- | --- | --- | --- | --- | --- |
| Agberotimi et al., 2020 (27) | Nigeria | Cross-sectional | Multiple cadres of HCWs during COVID-19 | Online | March-April 2020 | Online self-administered survey | Snowball sampling | 382 HCWs;  502 participants from the general population | Anxiety, depression, and PTSD | -Relatively few study participants may limit the generalizability of study findings.  -Self-reporting bias |
| Ahmed et al., 2023 (60) | Sudan | Cross-sectional | Multiple cadres of HCWs during COVID-19 | Hospital | Not reported. However, the initial manuscript was submitted for publication in December 2022 | Self-administered | Systematic sampling | 167 | Depression and anxiety | -Sample size may not be representative.  -Potential residual confounding. |
| Ali et al., 2021 (37) | Kenya | Cross-sectional | Nurses during COVID-19 | Online | August-November 2020 | Online self-administered survey | NR | 171 | Anxiety, Depression, and PTSD | -Study participants from one health facility limits generalizability to other health facilities.  -A smaller sample and shorter study duration may have led to smaller estimates.  -Potential selection bias and response bias. |
| Ali et el., 2022 (45) | Kenya | Cross-sectional | Doctors during COVID-19 | Online | August-November 2020 | Self-administered | Purposive sampling | 100 | Anxiety, depression, and PTSD | -Lack of comparative studies.  -Non-response bias.  -Could not conduct sub-group analysis. |
| Aliyu & Adeniyi, 2017 (28) | Nigeria | Cross-sectional | Multiple cadres of HCWs | Hospital | Not reported. However, the study was published in June 2017 | Self-administered | NR | 734 | Depression | -Self-report bias. |
| Ariyo et al., 2021 (61) | Nigeria | Cross-sectional | Multiple cadres of HCWs during COVID-19 | Hospital | October 2020-February 2021 | Self-administered | Purposeful sampling | 413 | Depression and anxiety | -Non-generalizability of study findings to the general population or other hospitals. |
| Arthur-Mensah et al., 2021 (62) | Ghana | Cross-sectional | Multiple cadres of HCWs during COVID-19 | Hospital | Not reported. However, the initial manuscript was submitted for publication in September 2021 | Self- & and interviewer-administered | Purposive sampling | 38 | State anxiety, trait anxiety, and depression | -Small sample size. |
| Asnakew et al., 2021 (56) | Ethiopia | Cross-sectional | Multiple cadres of HCWs during COVID-19 | Hospital | April-May 2020 | Self-administered | Simple random sampling | 396 | PTSD | -Cross-sectional design precludes causal inferences.  -Potential residual confounding.  -Lack of a valid PTSD tool. |
| Assefa et al., 2021 (23) | Burkina Faso  Ethiopia  Nigeria | Cross-sectional | Multiple cadres of HCWs during COVID-19 | Online | July-November 2020 | Interviewer-administered | NR | 900 | Anxiety and depression | -Potential selection bias.  -Limited generalizability of study findings to rural settings. |
| Ayalew et al., 2021 (63) | Ethiopia | Cross-sectional | Multiple cadres of HCWs during COVID-19 | Hospital | September-October 2020 | Self-administered | Simple random sampling | 387 | Anxiety and depression | -Use of screening tools rather than psychiatric interviews.  -Cross-sectional design precludes causal inferences.  -Non-face-to-face contact limited the collection of detailed information. |
| Ayalew et al., 2022 (57) | Ethiopia | Cross-sectional | Multiple cadres of HCWs during COVID-19 | Online | September-October 2020 | Self-administered | Simple random sampling | 387 | PTSD | -Use of screening tools rather than psychiatric interviews.  -Cross-sectional design precludes causal inferences.  -Non-face-to-face contact limited the collection of detailed information. |
| Bapolisi et al., 2022 (64) | Congo | Cross-sectional | Multiple cadres of HCWs during COVID-19 | Hospital | June-August 2020 | Self-administered | Purposive sampling | 252 | Depression and PTSD | -Cross-sectional design precludes causal inferences.  -Non-response bias.  -Social desirability bias.  -Limited generalizability to post-study periods or other settings. |
| Belayneh et al., 2021 (7) | Ethiopia | Cross-sectional | Nurses working in emergency and intensive care units | Hospital | April 2017 | Interviewer-administered | Systematic random sampling | 415 | Anxiety | -Cross-sectional design precludes causal inferences.  -Lack of a valid anxiety tool. |
| Belete & Anbensaw, 2022 (29) | Ethiopia | Cross-sectional | Multiple cadres of HCWs | Hospital | March 2020 | Self-administered | Stratified random sampling | 252 | Depression | -Cross-sectional design precludes causal inferences.  -Results might not be nationally representative. |
| Bernard et al., 2023 (30) | Nigeria | Cross-sectional | Early career doctors | Hospital | November 2019-January 2020 | Self-administered | Probability sampling | 629 | Anxiety | -Possible recall bias.  -Cross-sectional design precludes causal inferences. |
| Birhane et al., 2023 (31) | Ethiopia | Cross-sectional | Health extension workers | Health facility | March-May 2019 | Interviewer-administered | Random sampling | 1165 | Depression | -Cross-sectional design precludes causal inferences.  -Use of a non-validated tool. |
| Bundi et al., 2023 (65) | Kenya | Cross-sectional | Multiple cadres of HCWs during COVID-19 | Online | Not reported. However, the initial manuscript was submitted for publication in April 2023 | Self-administered | Stratified sampling | 202 | Depression and anxiety | -Potential non-response bias.  -Potential selection bias.  -Cross-sectional design precludes causal inferences. |
| Burnett-Zieman et al., 2023 (66) | Malawi | Cross-sectional | Multiple cadres of HCWs providing maternal healthcare during COVID-19 | Hospital | April-May 2021 | Interviewer-administered telephone survey | Purposeful sampling | 302 | Depression and PTSD | -Possible residual confounding.  -Possible social desirability bias.  -Possible model over-fit.  -Insufficient sample size. |
| Cenat et al., 2022 (15) | DRC | Cross-sectional | Multiple cadres of HCWs during COVID-19 | Hospital | October 2020 | Self-administered | Convenience sampling | 254 | Anxiety and  PTSD | -Cross-sectional design precludes causal inferences.  -Symptoms were not assessed pre- and post-pandemic, increasing the risk of recall bias.  -Use of tools covering a broad range of traumatic events. |
| Chorwe-Sungani, 2022 (38) | Malawi | Cross-sectional | Nurses during COVID-19 | Online | August-September 2020 | Self-administered | Purposive sampling | 102 | COVID-19 Anxiety | -Potential response bias.  -Self-report may have introduced reporter or recall bias. |
| Commander et al., 2020 (24) | Multicountry^1^ | Cross-sectional | Surgeons | Online | Not reported. However, the initial manuscript was submitted for publication in February 2020 | Online self-administered survey | NR | 131 | Depression | -Potential non-response bias.  -Possibility of reporting bias.  -Limited comparability of study findings to other settings.  -Trends over time of study outcomes not assessed. |
| Dawood et al., 2021 (67) | South Africa | Cross-sectional | Multiple cadres of HCWs during COVID-19 | Online | August-October 2020 | Self-administered | Convenience and snowball sampling | 312 | Anxiety, depression, and PTSD | -Close-ended questionnaires limited in-depth exploration.  -Potential bias due to the use of electronic data collection methods. |
| Duffton et al., 2023 (46) | South Africa | Cross-sectional | Frontline doctors during the COVID-19 pandemic | Online | November 2020-June 2021 | Self-administered | Purposeful sampling | 163 | Depression and anxiety | -Cross-sectional design precludes causal inferences.  -Limited generalizability across settings.  -Possible selection and response bias.  -Missing data may have led to loss of information. |
| Elamin et al., 2020 (68) | Sudan | Cross-sectional | Multiple cadres of HCWs during COVID-19 | Online | Not reported. However, the initial manuscript was submitted for publication in May 2020 | Self-administered | Stratified sampling | 396 | Depression, anxiety, and PTSD | -Potential selection bias.  -Cross-sectional design precludes causal inferences. |
| Falade et al., 2022 (69) | Nigeria | Cross-sectional | Multiple cadres of HCWs during COVID-19 | Online | March-July 2020 | Self-administered | Purposive sampling | 432 | Anxiety and depression | -The study was restricted to only participants who had smartphones and were literate. |
| GebreEyesus et al., 2021 (70) | Ethiopia | Cross-sectional | Multiple cadres of HCWs during COVID-19 | Hospital | November 2020 | Self-administered | Simple random sampling | 322 | Anxiety and depression | -Cross-sectional design precludes causal inferences.  -Self-report bias.  -Did not explore common risk factors for depression and anxiety. |
| Hain et al., 2021 (47) | South Africa | Cross-sectional | Doctors working in rural areas during COVID-19 | Hospital | August-September 2020 | Self-administered | Convenience sampling | 96 | Depression and anxiety | -Cross-sectional design precludes causal inferences.  -Potential selection and information bias.  -Limited generalizability of study findings. |
| Hajure et al., 2021 (71) | Ethiopia | Cross-sectional | Multiple cadres of HCWs during COVID-19 | Hospital | May 2020 | Self-administered | Convenience sampling | 127 | Depression, anxiety, and PTSD | -Convenience sampling limits generalizability.  -Potential residual confounding. |
| Hassan et al., 2024 (72) | Sudan | Cross-sectional | Multiple cadres of HCWs during COVID-19 | Hospital | September 2020-September 2021 | NR | NR | 385 | Depression and anxiety |  |
| Human et al., 2023 (58) | South Africa | Cross-sectional | Multiple cadres of HCWs providing mental healthcare during COVID-19 | Hospital | July 2021-June 2022 | Interviewer-administered | Simple random sampling | 120 | PTSD | -Cross-sectional design, small sample size, and specialized study population limit generalizability.  -Cross-sectional design precludes causal inferences. |
| Ibigbami et al., 2022 (42) | Nigeria | Cross-sectional | Doctors and nurses during COVID-19 | Hospital | July 2021 | Self-administered | Stratified sampling | 434 | Depression and anxiety | -Cross-sectional design precludes causal inferences.  -Potential recall bias.  -Limited generalizability to other cadres. |
| Idrees & Bashir, 2023 (73) | Sudan | Cross-sectional | Multiple cadres of HCWs during COVID-19 | Online | December 2020-January 2021 | Self-administered | Convenience sampling | 133 | Depression and anxiety | -Possible recall bias.  -Limited generalizability to other hospitals. |
| Jemal et al., 2022 (74) | Ethiopia | Cross-sectional | Multiple cadres of HCWs during COVID-19 | Hospital | June-July 2020 | Self-administered | Stratified sampling | 816 | Anxiety and depression | -Cross-sectional design precludes causal inferences.  -Limited generalizability to other settings. |
| Kabunga & Okalo, 2021 (39) | Uganda | Cross-sectional | Nurses during COVID-19 | Hospital | May-June 2021 | NR | Simple random sampling | 601 | PTSD | -Cross-sectional design precludes causal inferences.  -Potential response bias.  -Limited generalizability of study findings. |
| Kibret et al., 2020 (52) | Ethiopia | Cross-sectional | Multiple cadres of HCWs during COVID-19 | Hospital | May-June 2020 | NR | NR | 305 | Anxiety | -NR |
| Kim et al., 2019 (14) | Malawi | Cross-sectional | Multiple cadres of HCWs providing HIV care | Hospital | August 2015-January 2016 | Self-administered | Convenience sampling | 535 | Depression | -Potential social desirability bias. |
| Kwobah et al., 2021 (75) | Kenya | Cross-sectional | Multiple cadres of HCWs during COVID-19 | Online survey | April-June 2020 | Self-administered | Snowball sampling | 957 | Anxiety, depression, and PTSD | -Cross-sectional design precludes causal inferences.  -Potential response bias.  -Convenience sampling limits generalizability. |
| Mbanga et al., 2019 (4) | Cameroon | Cross-sectional | Nurses | Hospital | January-June 2018 | Self-administered | Consecutive sampling | 143 | Depression | -Potential recall bias.  -Overlap of symptoms and cross-sectional design precludes causal inferences. |
| Mc Magh et al., 2023 (76) | South Africa | Cross-sectional | Multiple cadres of HCWs during COVID-19 | Hospital | June 2021-March 2022 | Self-administered | NR | 330 | Depression, anxiety, and COVID-19 anxiety | - Cross-sectional design precludes causal inferences.  -Potential response bias.  -Potential recall bias.  -Potential social desirability bias. |
| Mekonen et al., 2021 (40) | Ethiopia | Cross-sectional | Nurses during COVID-19 | Hospital | September-October 2020 | Self-administered | Simple random sampling | 293 | Anxiety and depression | -Self-reporting bias.  -Short study duration.  -No sub-group analysis comparing nurses working in different departments. |
| Mokogwu, 2021 (77) | Nigeria | Cross-sectional | Multiples cadres of HCWs quarantined during COVID-19 | Home | April-May 2020 | Self-administered | NR | 32 | Depression and anxiety | -Small sample size.  -Potential response bias. |
| Mulatu et al., 2021 (78) | Ethiopia | Cross-sectional | Multiple cadres of HCWs during COVID-19 | Hospital | August 2020 | Self-administered | Simple random sampling | 420 | Anxiety, depression, and PTSD | -Findings might not be nationally representative.  -Cross-sectional design precludes causal inferences.  -Self-report may have introduced social desirability bias. |
| Muliira et al., 2015 (8) | Uganda | Cross-sectional | Midwives who have experienced maternal death | Hospital | Not reported. However, the study was published in 2015 | Self-administered | NR | 244 | Death anxiety | -Potential recall bias.  -Potential response bias.  -Limited generalizability to other settings.  -Potential selection bias. |
| Naidoo et al., 2020 (32) | South Africa | Cross-sectional | Doctors | Hospital | September 2018-January 2019 | Self-administered survey | Convenience sampling | 150 | Anxiety and depression | -Cross-sectional design precludes causal inferences.  -Potential bias due to self-report.  -Convenience sampling limits generalizability.  -Low response rate. |
| Nguepy Keubo et al., 2020 (79) | Cameroon | Cross-sectional | Multiple cadres of HCWs during COVID-19 | Online | April 2020 | Self-administered | Convenience sampling | 292 | Anxiety and depression | NR |
| Obi et al., 2015 (33) | Nigeria | Cross-sectional | Multiple cadres of HCWs | Hospital | Not reported. However, the study was published in 2015 | NR | Proportional quota sampling | 309 | Depression | -Cross-sectional design precludes causal inferences.  Possible residual confounding.  -Limited diagnostic validity of the depression scale used. |
| Ofori et al., 2021 (80) | Ghana | Cross-sectional | Multiple cadres of HCWs during COVID-19 | Hospital | July-August 2020 | Self-administered | Convenience sampling | 272 | Anxiety and depression | -Self-reporting bias.  -Limited generalizability to other settings.  -Absence of qualitative approaches that would have added more insights. |
| Oguntayo et al., 2022 (81) | Nigeria | Cross-sectional | Multiple cadres of HCWs during COVID-19 | Online | May-August 2021 | Self-administered | Snowball sampling | 300 | Anxiety, depression, and PTSD | -Potential response bias due to the use of online data collection methods.  -Potential underrepresentation of some sub-populations due to the snowball sampling.  -No direct access to study participants due to COVID-19. |
| Olabisi et al., 2021 (34) | Nigeria | Cross-sectional | Nurses | Hospital | Not reported. However, the initial manuscript was submitted for publication in February 2021 | Self-administered | Convenience sampling | 360 | Anxiety and depression | -Cross-sectional design precludes causal inferences.  -Potential response bias. |
| Olashore et al., 2018 (35) | Botswana | Cross-sectional | Multiple cadres of HCWs providing mental care | Hospital | August-November 2016 | Self-administered | NR | 201 | PTSD | -Potential response and recall bias.  -Cross-sectional design precludes causal inferences.  -Potential residual confounding. |
| Olashore et al., 2021 (25) | Botswana  Nigeria | Cross-sectional | Multiple cadres of HCWs during COVID-19 | Hospital | May-September 2020 | Self-administered | NR | 373 | Anxiety | -Cross-sectional design precludes causal inferences.  -Small sample size limited the generalizability of findings.  -Limited interpretability of findings due to the use of non-culturally adapted tools.  -Self-report may have introduced bias. |
| Olashore et al., 2022 (82) | Botswana | Cross-sectional | Multiple cadres of HCWs during COVID-19 | Hospital | June-October 2020 | Self-administered survey | Convenience sampling | 353 | Anxiety and depression | -Cross-sectional design precludes causal inferences.  -Potential under- or over-reporting due to the use of elf-reporting tools.  -PTSD not assessed.  -Inability to sample all health facilities limits generalizability. |
| Onchonga et al., 2021 (83) | Kenya | Cross-sectional | Multiple cadres of HCWs during COVID-19 | Online | Not reported. However, the initial manuscript was submitted for publication in September 2020 | Self-administered | Stratified sampling | 476 | Anxiety and depression | -Study only limited to depression and anxiety. |
| Osasona & Oderinde, 2023 (54) | Nigeria | Cross-sectional | Multiple cadres of HCWs during COVID-19 | Hospital | May-July 2021 | Self-administered | Convenience sampling | 213 | Anxiety | A small sample size and few study settings limit generalizability. |
| Phiri et al., 2023 (50) | Malawi | Cross-sectional | Multiple cadres of HCWs providing HIV care during COVID-19 | Hospital | April-May 2021 | Interviewer-administered | Purposeful and random sampling | 435 | Depression | -Possible social desirability bias.  -Cross-sectional design precludes causal inferences.  -Limited generalizability of study findings.  -Lack of comparison data across COVID-19 waves. |
| Pindar et al., 2015 (36) | Nigeria | Cross-sectional | Multiple cadres of HCWs | Hospital | Not Reported | Self-administered | Stratified sampling | 186 | Depression | -Cross-sectional design precludes causal inferences. |
| Quadri et al., 2021 (26) | Multicounty^2^ | Cross-sectional | Multiple cadres of HCWs during COVID-19 | Online | April-May 2020 | Self-administered | Snowball sampling | 489 | Depression | -Potential risk of bias.  -No formal power analysis.  -Snowball sampling.  -Potential residual confounding. |
| Sagaon-Teyssier et al., 2020 (84) | Mali | Cross-sectional | Multiple cadres of HCWs providing HIV care during COVID-19 | Hospital | April 2020 | Self-administered | NR | 135 | Depression and anxiety | -Possible residual confounding due to small sample size.  -Non-generalizability of study findings.  -Short questionnaire prevented the collection of more detailed information. |
| Shah et al., 2021 (43) | Kenya | Cross-sectional | Doctors and nurses during COVID-19 | Online | August-November 2020 | Self-administered | Purposive sampling | 433 | Anxiety, depression, and PTSD | -Cross-sectional design precludes causal inferences.  -Potential non-generalizability of findings.  -Potential response bias. |
| Shumye et al., 2022 (6) | Ethiopia | Cross-sectional | Multiple cadres of HCWs during COVID-19 | Online | May-June 2020 | Self-administered | Random sampling | 207 | PTSD and depression | -Cross-sectional design precludes causal inferences.  -Small sample size limits generalizability.  -Use of self-report screening tools instead of psychiatric interviews. |
| Siamisang et al 2022 (85) | Botswana | Cross-sectional | All frontline HCWs during COVID-19 | Hospital | July-September 2021 | Self-administered | Purposive sampling | 447 | Depression and anxiety | -Potential selection bias.  -Potential residual confounding.  -Limited comparability of study findings to other studies.  -Cross-sectional design may have led to underreporting. |
| Simbeza et al., 2023 (51) | Zambia | Cross-sectional | Multiple cadres of HCWs during COVID-19 | Hospital | August-October 2020 | Self- or interviewer-administered | Convenience sampling | 713 | Depression | -Possible residual confounding.  -Convenience sampling limits the generalizability of study findings.  -Lack of pre-COVID-19 comparison data. |
| Teshome et al., 2020 (53) | Ethiopia | Cross-sectional | Multiple cadres of HCWs during COVID-19 | Hospital | May-June 2020 | Interviewer-administered survey | Simple random sampling | 798 | Anxiety | -Cross-sectional design precludes causal inferences.  -Social desirability bias. |
| Vancampfort et al., 2022 (41) | Uganda | Cross-sectional | Mental health nurses during COVID-19 | Hospital | January-March 2021; September-November 2021 | Interviewer-administered | Cluster sampling | 108 | PTSD | -Cross-sectional design precludes causal inferences.  -Self-report may have introduced reporting bias. |
| Wayessa et al., 2021 (48) | Ethiopia | Cross-sectional | Multiple cadres of HCWs during COVID-19 | Hospital | June-July 2020 | Self-administered | Simple random sampling | 275 | Depression | -Cross-sectional design precludes causal inferences.  -Potential reporting bias due to self-report measures. |
| Wayessa et al., 2023 (55) | Ethiopia | Cross-sectional | Multiple cadres of HCWs during COVID-19 | Hospital | June-July 2020 | Self-administered | Simple random sampling | 275 | Anxiety | - Cross-sectional design precludes causal inferences.  -Potential residual confounding.  -Potential recall bias. |
| Workneh et al., 2023 (44) | Ethiopia | Cross-sectional | Doctors and nurses during COVID-19 | Online | September 2020; October 2021 | Interviewer-administered telephone survey | Simple random sampling | 577 | Depression | A small sample size and the selection criteria limit the generalizability of study findings.  -Possible social desirability bias.  -Possible residual confounding. |
| Yadeta et al., 2021 (49) | Ethiopia | Cross-sectional | Multiple cadres of HCWs during COVID-19 | Hospital | October-November 2020 | Self-administered | Random sampling | 265 | Depression | -Cross-sectional design precludes causal inferences.  -Potential unaccounted and residual confounding.  -Only one outcome was assessed. |
| Yitayih et al., 2020 (59) | Ethiopia | Cross-sectional | Multiple cadres of HCWs during COVID-19 | Hospital | March 2020 | Self-administered | Systematic sampling | 249 | PTSD | -Non-generalizability of study findings.  -Potential social desirability bias.  -Cross-sectional design precludes causal inferences. |
| 1- Botswana, Burundi, Cameroon, Democratic Republic of Congo, Ethiopia, Gabon, Ghana, Kenya, Malawi, Mozambique, Namibia, Rwanda, Sudan, Tanzania, Zambia, Zimbabwe  2- Egypt, Ethiopia, Kenya, Nigeria, Somalia, Sudan, Tanzania, Uganda, Malawi, Sierra Leone, Rwanda, South Sudan, Gambia  PTSD – Posttraumatic Stress Disorder’; COVID-19 – 2019 Corona Virus Disease; HCWs – Healthcare Workers; NR – Not Reported | | | | | | | | | | |
