# Supplementary material for: Prevalence and factors associated with depression, anxiety and post-traumatic stress disorder among healthcare workers from sub-Saharan Africa: systematic review
Source: BJPsych Open. 2025 Sep 8;11(5):e192. doi: 10.1192/bjo.2025.10818 (PMC12451537; doi:10.1192/bjo.2025.10818)
Supplement: Too et al. supplementary material 3 — Too et al. supplementary material [file S2056472425108181sup003.docx]

| **First author (publication year)** | **Selection of participants** | | | | **Comparability** | **Ascertainment of Outcome** | | **Total score** | **Quality grade** |
| --- | --- | --- | --- | --- | --- | --- | --- | --- | --- |
|  | **Sample representativeness** | **Sample size** | **Non-respondence rate** | **Screening tool ascertainment** | **Confounders investigated** | **Outcome assessment** | **Statistical test appropriateness** |  |  |
| Ahmed et al., 2023 (60) | * | - | - | * | - | * | - | 3 | Unsatisfactory |
| Agberotimi et al., 2020 (27) | * | * | - | ** | * | * | * | 7 | Good |
| Ali et al., 2021 (37) | - | - | - | ** | * | * | * | 5 | Satisfactory |
| Ali et al., 2022 (45) | - | - | - | ** | * | * | * | 5 | Satisfactory |
| Aliyu & Adeniyi., 2017 (28) | - | - | - | * | * | * | * | 4 | Unsatisfactory |
| Ariyo et al., 2021 (61) | * | - | - | ** | * | * | * | 6 | Satisfactory |
| Arthur-Mensah et al., 2022 (62) | - | - | - | * | * | * | * | 4 | Unsatisfactory |
| Asnakew et al., 2021 (56) | * | * | * | ** | * | * | * | 8 | Good |
| Assefa et al., 2021 (23) | * | * | - | * | * | * | * | 6 | Satisfactory |
| Ayalew et al., 2021 (57) | * | * | - | ** | * | * | * | 7 | Good |
| Ayalew et al., 2021 (63) | * | * | - | ** | * | * | * | 7 | Good |
| Bapolisis et al., 2022 (64) | * | - | - | * | * | * | * | 5 | Satisfactory |
| Belayneh et al., 2021 (7) | * | * | * | * | * | * | * | 7 | Good |
| Belete et al., 2022 (29) | * | * | * | ** | * | * | * | 8 | Good |
| Bernard et al., 2023 (30) | * | * | - | ** | * | * | * | 7 | Good |
| Birhane et al., 2023 (31) | * | * | - | * | * | * | * | 6 | Satisfactory |
| Bundi et al., 2023 (65) | * | * | - | ** | * | * | * | 7 | Good |
| Burnett-Zieman et al., 2023 (66) | * | * | - | ** | * | * | * | 7 | Good |
| Cénat et al., 2022 (15) | * | * | - | ** | * | * | * | 7 | Good |
| Chorwe-Sungani, 2021 (38) | * | * | - | ** | * | * | * | 7 | Good |
| Commander et al., 2020 (24) | - | * | - | * | * | * | * | 5 | Satisfactory |
| Dawood et al., 2022 (67) | * | * | - | * | * | * | * | 6 | Satisfactory |
| Duffton et al., 2023 (46) | * | * | - | ** | * | * | * | 7 | Good |
| Elamin et el., 2020 (68) | * | * | - | ** | * | * | * | 7 | Good |
| Falade et al., 2022 (69) | * | * | - | * | * | * | * | 6 | Satisfactory |
| GebreEyesus et al., 2021 (70) | * | * | * | * | * | * | * | 7 | Good |
| Hain et al., 2021 (47) | * | - | - | * | * | * | * | 5 | Satisfactory |
| Hajure et al., 2021 (71) | * | - | - | * | * | * | * | 5 | Satisfactory |
| Hassan et al., 2023 (72) | - | - | - | * | * | * | * | 4 | Unsatisfactory |
| Human et al., 2023 (58) | * | * | - | * | * | * | * | 6 | Satisfactory |
| Ibigbami et al., 2022 (42) | * | * | - | ** | * | * | * | 7 | Good |
| Idrees & Bashir, 2023 (73) | * | * | - | * | * | * | * | 6 | Satisfactory |
| Jemal et al., 2021 (74) | * | * | * | ** | * | * | * | 8 | Good |
| Kabunga & Okalo, 2021 (39) | * | * | - | ** | * | * | * | 7 | Good |
| Kibret et al., 2020 (52) | - | - | - | ** | * | * | * | 5 | Satisfactory |
| Kim et al., 2019 (14) | * | - | * | ** | * | * | * | 7 | Good |
| Kwobah et al., 2021 (75) | * | * | - | * | * | * | * | 6 | Satisfactory |
| Mbanga et al., 2019 (4) | * | - | * | * | * | * | * | 6 | Satisfactory |
| Mc Magh et al., 2023 (76) | - | * | - | * | * | * | * | 5 | Satisfactory |
| Mekonen et al., 2020 (40) | * | * | * | * | * | * | * | 7 | Good |
| Mokogwu, 2021 (77) | - | - | - | * | - | * | * | 3 | Unsatisfactory |
| Mulatu et al., 2021 (78) | * | * | * | ** | * | * | * | 8 | Good |
| Muliira et al., 2015 (8) | * | * | * | * | * | * | * | 7 | Good |
| Naidoo et al., 2020 (32) | * | - | - | * | * | * | * | 5 | Satisfactory |
| Nguepy Keubo et al., 2020 (79) | * | * | - | ** | * | * | * | 7 | Good |
| Obi et al., 2015 (33) | * | * | - | * | * | * | - | 5 | Satisfactory |
| Ofori et al., 2021 (80) | * | * | - | * | * | * | * | 6 | Satisfactory |
| Oguntayo et al., 2022 (81) | * | - | - | ** | * | * | * | 6 | Satisfactory |
| Olabisi et al., 2022 (34) | * | * | - | * | - | * | * | 5 | Satisfactory |
| Olashore et al., 2018 (35) | * | - | * | * | * | * | * | 6 | Satisfactory |
| Olashore et al., 2021 (25) | * | * | - | ** | * | * | * | 7 | Good |
| Olashore et al., 2022 (82) | * | * | * | * | * | * | * | 7 | Good |
| Onchonga et al., 2022 (83) | * | * | - | * | * | * | * | 6 | Satisfactory |
| Osasona & Oderinde, 2023 (54) | * | * | - | ** | * | * | * | 7 | Good |
| Phiri et al., 2023 (50) | * | * | - | * | * | * | * | 6 | Satisfactory |
| Pindar et al., 2020 (36) | * | * | - | * | * | * | * | 6 | Satisfactory |
| Quadri et al., 2021 (26) | * | * | - | * | * | * | * | 6 | Satisfactory |
| Sagaon-Teyssier et al., 2023 (84) | - | - | - | * | * | * | * | 4 | Unsatisfactory |
| Shah et al., 2021 (43) | * | - | ­- | ** | * | * | * | 6 | Satisfactory |
| Shumye et al., 2022 (6) | * | * | - | * | * | * | * | 6 | Satisfactory |
| Siamisang et al., 2020 (85) | * | * | - | ** | * | * | * | 7 | Good |
| Simbeza et al., 2023 (51) | * | - | - | * | * | * | * | 5 | Satisfactory |
| Teshome et al., 2020 (53) | * | * | * | ** | * | * | * | 8 | Good |
| Vancampfort et al., 2022 (41) | * | * | * | * | * | * | * | 7 | Good |
| Wayessa et al., 2021 (48) | * | * | * | * | * | * | * | 7 | Good |
| Wayessa et al., 2023 (55) | * | * | * | ** | * | * | * | 8 | Good |
| Workneh et al., 2023 (44) | * | - | - | ** | * | * | * | 6 | Satisfactory |
| Yadeta et al., 2021 (49) | * | * | - | ** | * | * | * | 7 | Good |
| Yitayih et al., 2020 (59) | * | - | * | ** | * | * | * | 7 | Good |
